# Multiple Wavelength Photopolymerization of Stable Poly(Catecholamines)‐DNA Origami Nanostructures**

**DOI:** 10.1002/anie.202111226

**Published:** 2022-01-03

**Authors:** Pia Winterwerber, Colette J. Whitfield, David Y. W. Ng, Tanja Weil

**Affiliations:** ^1^ Max Planck Institute for Polymer Research Ackermannweg 10 55128 Mainz Germany

**Keywords:** Cell Uptake, DNA Origami, Multiwavelength, Photopolymerization, Poly(Catecholamines)

## Abstract

The synthesis of multicomponent polymer hybrids with nanometer precision is chemically challenging in the bottom‐up synthesis of complex nanostructures. Here, we leverage the fidelity of the DNA origami technique to install a multiple wavelength responsive photopolymerization system with nanometer resolution. By precisely immobilizing various photosensitizers on the origami template, which are only activated at their respective maximum wavelength, we can control sequential polymerization processes. In particular, the triggered photosensitizers generate reactive oxygen species that in turn initiate the polymerization of the catecholamines dopamine and norepinephrine. We imprint polymeric layers at designated positions on DNA origami, which modifies the polyanionic nature of the DNA objects, thus promoting their uptake into living cells while preserving their integrity. Our herein proposed method provides a rapid platform to access complex 3D nanostructures by customizing material and biological interfaces.

## Graphical abstract

DNA origami nanostructures have been decorated with photosensitive reaction centers that produce reactive oxygen species at their individual characteristic wavelengths. In this way, polymerization of the catecholamines dopamine and norepinephrine is induced and independent polymer patterning on the DNA origami template can be directed. In addition, the polymer rings alter the interaction of DNA origami objects and cell membranes, thereby promoting their cellular uptake.

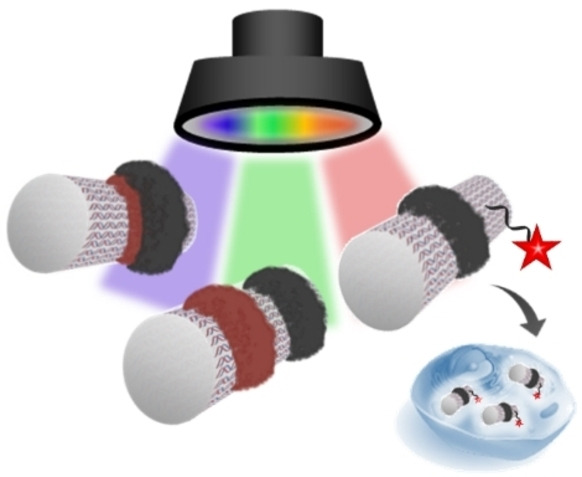

## Introduction

The spatial control and engineering of objects at nanometer resolution is imperative for the miniaturization of smart materials and devices. In both materials science and biomedicine, the demand for tools to construct multicomponent substructures across 3D space is required to expand the understanding of how surface patterns and object contours modulate interfacial forces. However, the construction of nanostructured surfaces on soft materials that can be freely customized is a bottleneck due to the lack of tools to precisely design them. This problem is further amplified for patterns that are much smaller than the wavelength of light, where top‐down approaches, such as lithography, reaches its limits. At this length scale, bottom‐up approaches based on self‐assembly provide the natural complementarity to top‐down strategies in the fabrication of patterned soft materials.

Unlike the limitations posed by other systems based on synthetic polymers or peptides, DNA nanotechnology is equipped with the precision necessary to program nanostructured surfaces.^[1]^ Coupled with a DNA origami design,^[2]^ concepts to investigate epitopes,^[3]^ protein assemblies,^[4]^ plasmonic devices,^[5]^ and biosensing^[6]^ have recently resulted in critical findings in nanomedicine and biophysics.

In polymer chemistry and patterning, advances in radical and oxidative polymerization as well as the organization of polymer chains have demonstrated that the stringent conditions necessary for DNA origami can be made accessible to largely organic compounds.^[7]^ Conversely, the combination of DNA nanostructures with charged molecules and polymers has resulted in increased stability under physiological conditions and even in organic solvents, which has been crucial for the rapid expansion of DNA origami platforms in recent years.^[8]^ However, in comparison to the application‐driven counterparts, polymers on DNA origami have yet to show their synthetic potential beyond structured positioning by DNA hybridization on the template.^[9]^

**Figure 1:**
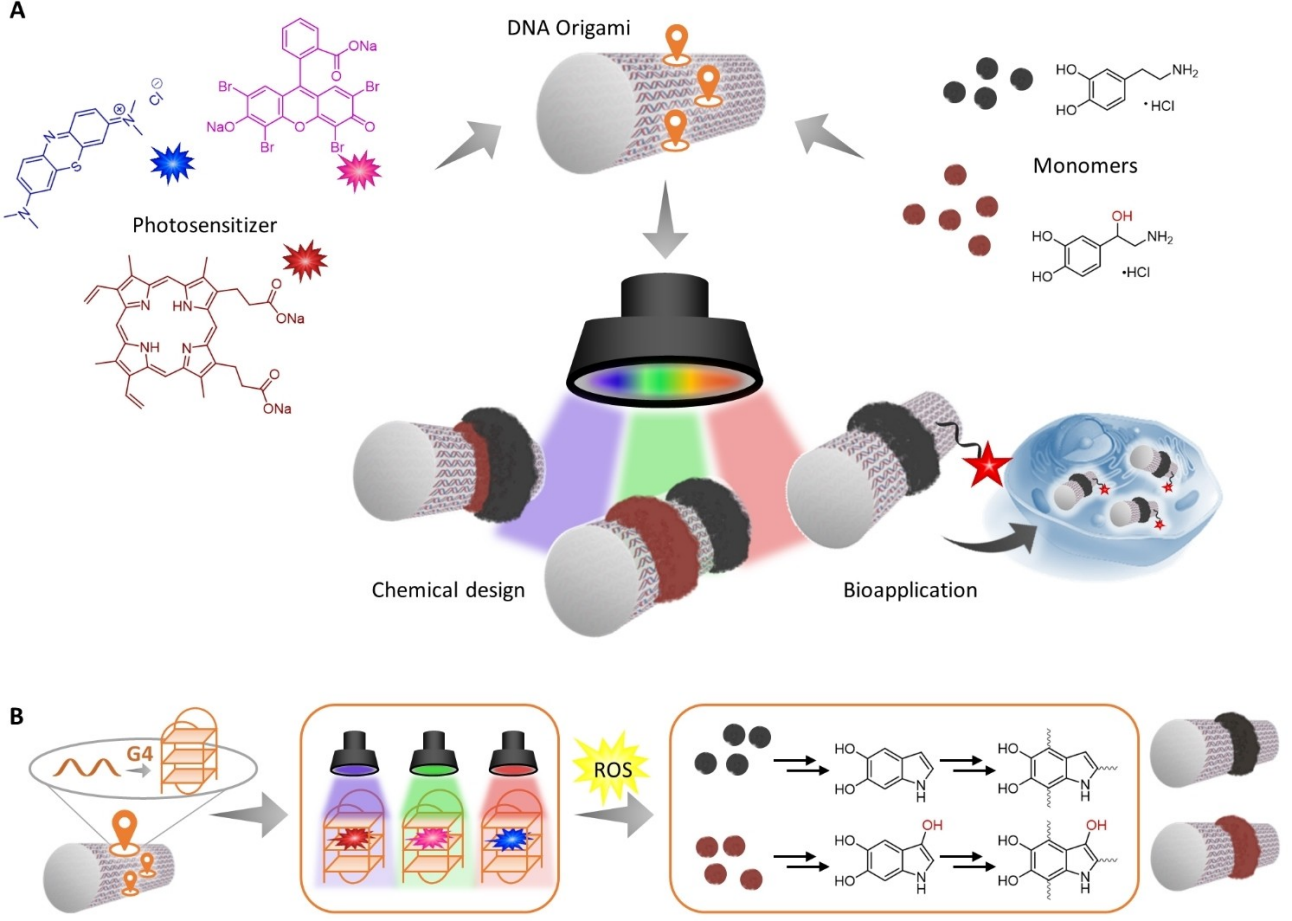
A) Multiple wavelength photopolymerization on DNA origami tubes can be accomplished through the combination of various photosensitizers and two different catecholamine monomers. In this way, polymers can be imprinted at specific sites on the DNA templates under temporal and spatial control. Polymer‐DNA hybrid structures can be further leveraged to modulate interactions at the cellular interface. B) The reaction centers, consisting of G‐quadruplex (G4) structures and photosensitizers, produce reactive oxygen species (ROS) at their maximum wavelengths to initiate polymerization. The mechanism and structure of both polydopamine^[11]^ and norepinephrine^[12]^ are multifaceted and still the object of current elucidation. For reasons of clarity, only a few representative structures are depicted here. Further information on the mechanism, the intermediates, and prevailing interactions is provided in the Supporting Information (Figure S2).

In this study, we control a series of photopolymerization reactions using multiple wavelengths to guide independent polymer patterns and fabricate layered structures on the DNA origami (Figure 1). Previously, we have shown that dopamine (DA) can be photopolymerized by protoporphyrin IX (PPIX) that is intercalated into a DNA G‐quadruplex (G4), which allows polydopamine to form at designated positions preoccupied by the G4.^[10]^ Herein, we establish broad wavelength flexibility by using G4s containing eosin Y (EY) and methylene blue (MB), which are activated by green (525 nm) and red (625 nm) light, respectively. Together with blue‐light‐triggered PPIX (410 nm), these three catalyst centers produce reactive oxygen species (ROS) at their maximum wavelengths to initiate the polymerization of catecholamines. By using both dopamine and norepinephrine (NE), we demonstrate that both monomers can be polymerized sequentially in different configurations, thereby allowing DNA‐polymer structures to be customized in the *z*‐direction. The formation of a polymer layer reduces the polyanionic nature of the DNA origami and can thereby facilitate its uptake into living cells, which can be imaged by fluorescence colocalization. Our approach will enable rapid and facile synthesis of multicomponent polymeric patterns with precise shapes and dimensions on DNA origami. Customizing the nano/bio‐interphase of DNA objects through surface modulation is crucial for various applications, for example, cellular uptake for therapeutic delivery.

## Results and Discussion

To prepare 3D DNA origami tubes for photopolymerization, G‐quadruplex structures were arranged on the surface in distinct patterns. These catalytic centers can be tuned for wavelength selectivity by nominating the photosensitizer that will sit within the G‐quadruplex (5′‐GGG TA GGG C GGG TT GGG‐3′). As previously reported, the PPIX‐G4 complex produces ROS under irradiation with white light, which in turn trigger the oxidation and polymerization of dopamine. To suppress the well‐known self‐polymerization of dopamine and to control polymer formation, it is crucial to work in a slightly acidic environment (pH 6.5). Herein, we found that only blue light (410 nm) possesses sufficient energy to initiate the polymerization of dopamine (see Figure S1 in the Supporting Information). Excitation at the Q‐bands of PPIX‐G4 in the visible spectrum produced insufficient oxidized dopamine species to fuel polymerization. Wavelength specificity in the green and red region was accomplished by hosting EY^[13]^ and MB,^[14]^ respectively, within the G4 motif. The propensity of each catalyst to generate singlet oxygen (^1^O_2_) was analyzed using an assay based on imidazole and *p*‐nitrosodimethylaniline (RNO).^[15]^ Both MB and EY demonstrated higher efficiencies than PPIX in the production of ^1^O_2_, as reflected by the bleaching of RNO being 7 and 11 times faster, respectively (see Figure S3 in the Supporting Information). The initiation of polymerization using EY‐G4 and MB‐G4 on a tube DNA origami was subsequently attempted at their respective wavelengths (EY: 525 nm, MB: 625 nm). The tube DNA origami scaffold was designed with a central ring containing photosensitizer‐loaded G4 sequences. When using 10 mm dopamine in 100 mm buffer (pH 6.5), UV/Vis spectroscopy showed successful polymerization into polydopamine (pDA) after 3 h (see Figure S4A in the Supporting Information). The formation of intermediates including dopaminochrome (320 nm), oxidized oligomers (480 nm), and the eventual pDA (700 nm) could be monitored through their characteristic absorbances.^[7c]^ Spatial control over polymerization and the resulting nanostructure were verified by atomic force microscopy (AFM; see Figure S4B in the Supporting Information). A ring of polymers was successfully constructed where the patterned G4 sequences were installed, thus demonstrating that the change of catalytic centers and excitation wavelengths did not affect the control over the polymerization reaction. In comparison, the reaction kinetics of the oxidative polymerization demonstrated that the generation of each intermediate (dopaminochrome, oligomers etc.) including pDA was more efficient for EY (see Figure S4C in the Supporting Information). Despite the differences in kinetics, topological height profile analysis by AFM did not show significant height differences between the different photosensitizers (see Figure S4D in the Supporting Information ).

**Figure 2:**
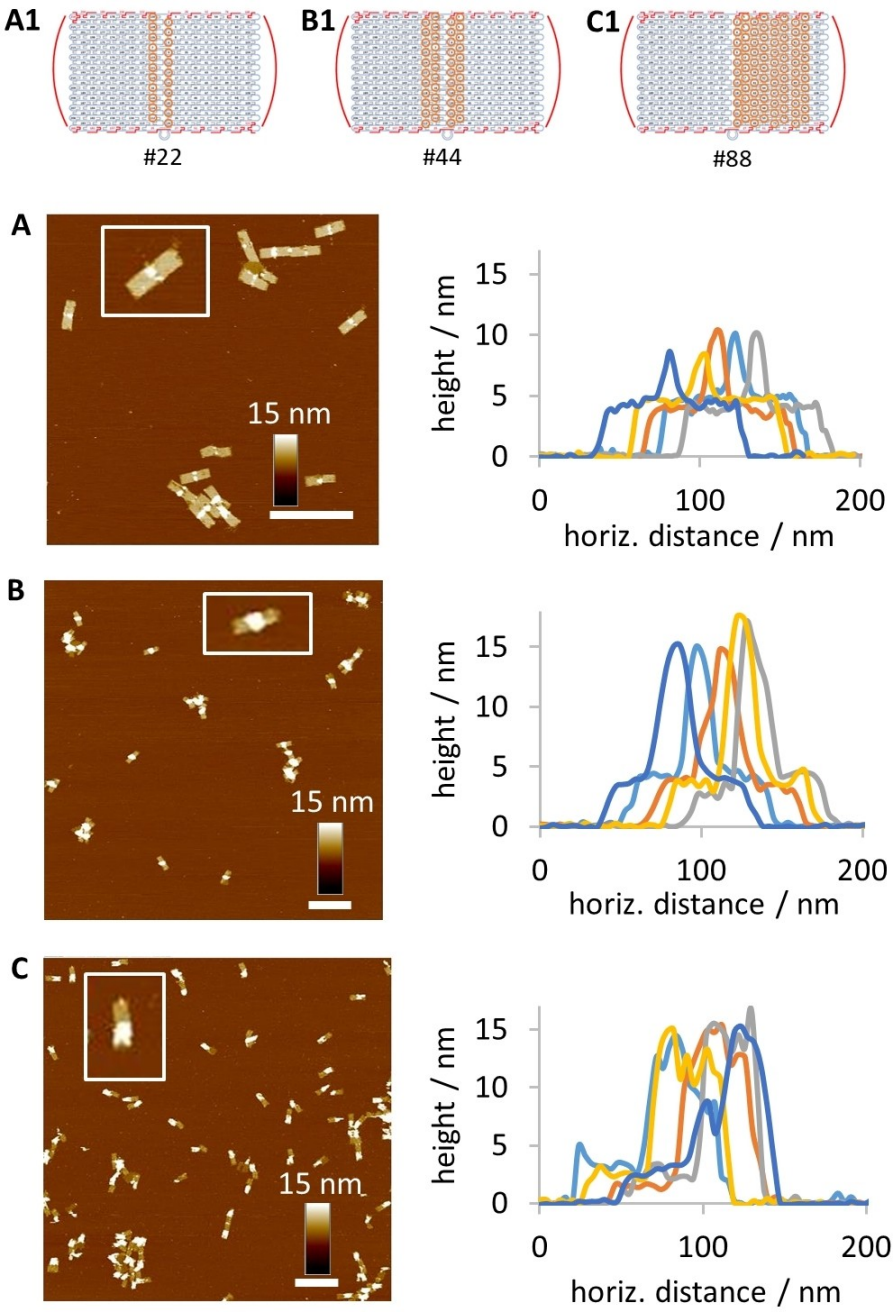
Studies on the correlation of polymer formation and the number of catalytic G4 centers. (A1–C1) DNA origami tubes were designed bearing a ring of 22, 44, or 88 G4 sequences, respectively. Tubes are rolled up by annealing the folding strands (depicted in red). A–C) AFM topographical images of the pDA‐ringed origami tubes reveal that a minimal number of 44 catalytic centers is required to reliably induce polymerization.

We subsequently investigated different patterns and the impact of the size of clustered G4 centers on the polymerization process. Firstly, we designed an origami tube with a diagonal G4 motif of similar density to the standard ring pattern (see Figure S5 in the Supporting Information). After polymerization, we could detect formation of pDA on the tubes, with varying observation perspective of the designated pattern. The lack of symmetry would mean that the orientation of the origami on the mica surface is subjected to inherent randomness and thus affects the imaging process. Hence, we consider ring patterns as the most reliable to provide robust characterization. In addition, we examined the correlation of polymer formation and the width of the ring system by direct comparison of the structures depicted in Figure 2. AFM images indicate that, when only 22 catalytic centers are incorporated, the oxidative conditions are not sufficient to induce polymerization on each tube. In addition, the thickness of the observable polymer rings is lower compared to the standard origami tube (44 G4). Here, total heights of typically 10–15 nm are attained. When doubling the number of G4 to 88, polymer rings are uniformly grown on almost every origami and the overall heights are similar to the standard 44 G4s. Our findings suggest that there is indeed a minimal number of clustered catalytic centers required to induce polymerization. Regarding the activity of these centers, no upper limit seems to restrict the system. However, full coverage of the tube's surface with G4 sequences would on the one hand restrict further modifications and on the other hand may harm the origami's integrity due to higher tensions in the rolling up process of the tube.

**Figure 3:**
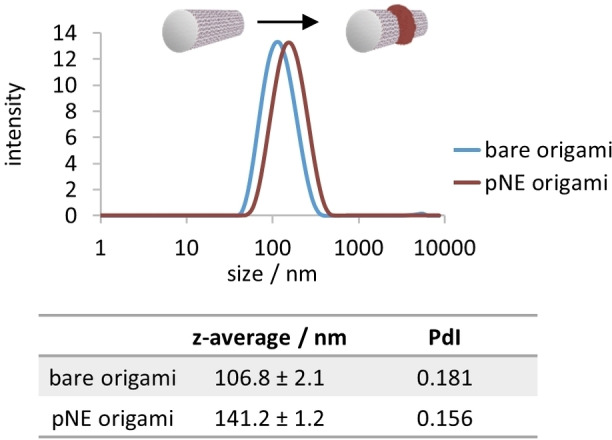
DLS characterization of bare origami tubes and pNE‐ringed origami tubes. Polymer growth on the origami causes a significant shift in the intensity‐weighted size distribution and *z*‐average values. The numbers should only be considered as a qualitative indication, since DLS operates on the principles of spherical objects, which does not apply that well for these origami structures.

Throughout the experiments it was noted that the polymer‐ringed DNA origami tubes tend to aggregate due to the strong adhesiveness of pDA (see Figure S6 in the Supporting Information). Therefore, norepinephrine was introduced as a dopamine analogue to achieve well‐dispersible nanoobjects that also remain stable in complex media without aggregate formation. Norepinephrine also belongs to the catecholamine family and poly(norepinephrine) (pNE) reveals material‐independent modification capabilities similar to pDA but with an ultrasmooth surface morphology.[^12c^, ^16^] Chemically, NE possesses an additional hydroxy group and this increase in hydrophilic interactions could potentially prevent aggregation of the formed nanostructures. In contrast to pDA, polymerization to pNE using all three photosensitizers showed a strong preference toward EY and MB (see Figure S7 in the Supporting Information). Observations from UV/Vis spectroscopic analysis suggested that the oxidation of pNE requires a higher performance photosensitizer to fuel the polymerization reaction. Likewise, the eventual formation of pNE on the DNA origami showed that the polymerization was more efficient with EY and MB as the photosensitizer. In dynamic light scattering (DLS) studies, the formation of a pNE ring caused a clear shift of the intensity‐based size distribution towards higher hydrodynamic diameters, which can also be seen in the *z*‐average values (Figure 3). Moreover, both bare origami and pNE‐ringed origami are stable for at least three days and do not show agglomeration (see Figure S8 in the Supporting Information).

Additionally, agarose gel electrophoresis (AGE) supplements the characterization of the origami tubes before and after polymerization of the pDA and pNE polymers (see Figure S9 in the Supporting Information).

**Figure 4:**
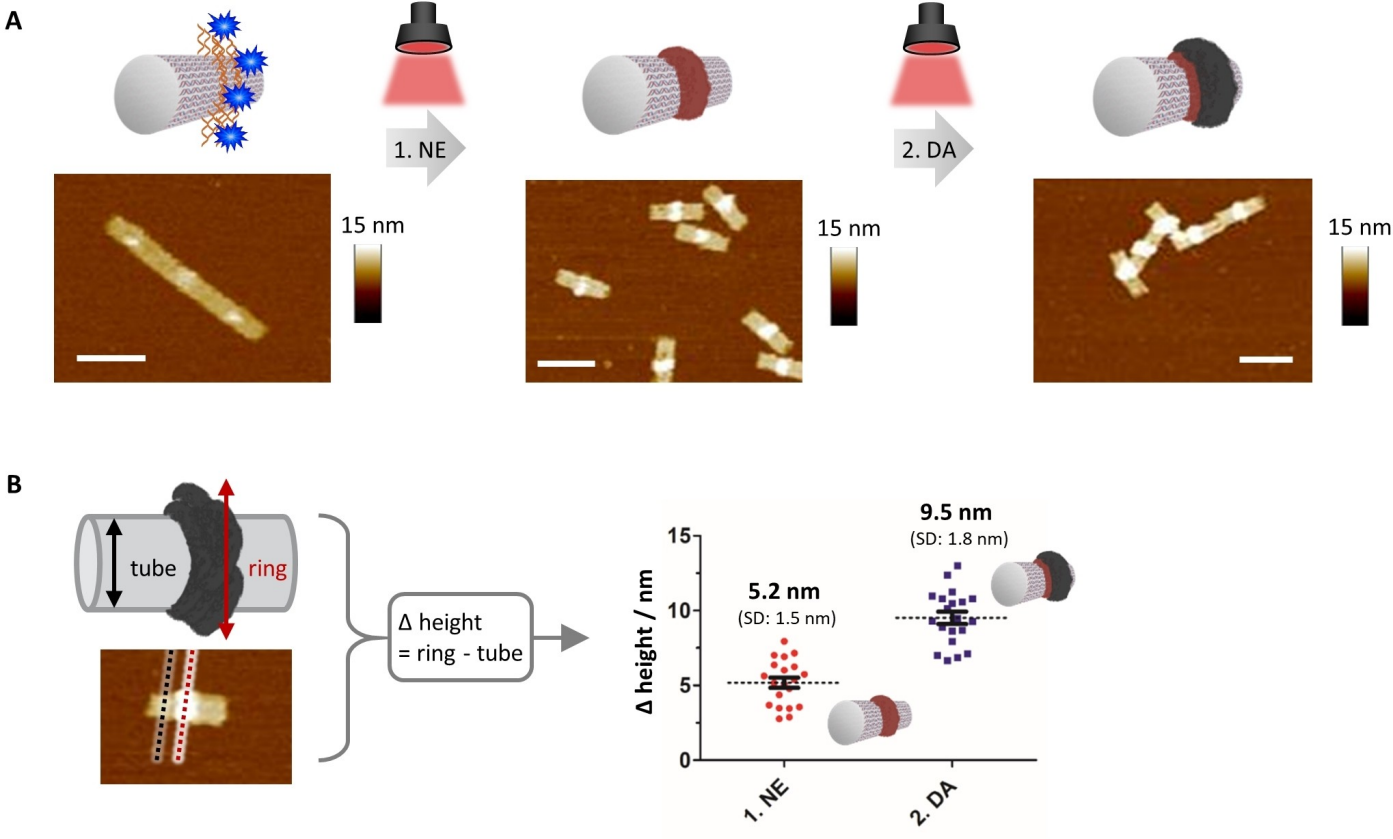
A two‐step polymerization process demonstrates the potential of the origami system to induce a layer‐by‐layer formation of polymers. A) DNA origami tubes are incubated with methylene blue as the photosensitizer and irradiated at 625 nm. In the first illumination phase, norepinephrine (NE) is polymerized, followed by a purification step to remove remaining NE, and dopamine (DA) is added as the second monomer that also polymerizes under irradiation with red light. AFM images show the topographical profile of the DNA origami tubes. The polymer is imprinted on top of the G4 patterns. Scale bars are 100 nm. B) To quantity the thickness of the polymer layers, *z*‐value heights were recorded and calculated as depicted (*n*=20; error bars are SEM).

Based on the acquired reaction conditions and wavelength selectivities pertaining to DA and NE, sequential polymerization steps were performed to fabricate multicomponent nanostructures. The DNA origami tubes were loaded with MB‐G4s, and NE and DA were sequentially polymerized at 625 nm for 2 h each (Figure 4A). Successful polymerization was detected for both illumination phases, giving the characteristic profiles for pNE and pDA (see Figure S10 in the Supporting Information). The first irradiation phase resulted in the formation of a pNE layer of 5.2 nm±1.5 nm (Figure 4B). Thereafter, excess NE and oxidized side products were removed by spin filtration and replaced by DA. The second irradiation phase yielded the pDA layer that contributed an additional height increase of 4.3 nm±1.8 nm (total height: 9.5 nm±1.8 nm; Figure 4B). The contributed height increase of each component correlates well to their individual single polymerizations. To demonstrate that the second irradiation phase had initiated the polymerization of DA and not the existing pNE (or its adsorbed oligomers), a control experiment was performed without the addition of DA. In this case, no additional polymers were formed (see Figure S11in the Supporting Information). The upper limit of the layered components is dictated by the access of monomers to the catalytic centers. At a total height of approximately 10–15 nm, the polymerization can no longer be guided by the photosensitizer‐G4 complex.

Next, we demonstrated wavelength orthogonality for a two‐step polymerization to achieve reaction selectivity. The dormancy of MB and EY at opposing wavelengths was first investigated. MB showed no generation of the oxidized species of DA when irradiated at 525 nm, and, likewise, EY was not active at 625 nm (see Figure S12 in the Supporting Information). To ensure that a consecutive activation of each photosensitizer can continuously trigger the polymerization of dopamine, the DNA origami tubes were loaded with ring patterns of EY‐G4 and MB‐G4 at each tubular end. Polymerization was initiated with DA (10 mm, pH 6.5) by sequential irradiation at 625 nm and 525 nm for 3 h each. The generation of oxidized intermediates and pDA was verified by UV/Vis spectroscopy (see Figure S13A in the Supporting Information). Control experiments illustrated that the integrity of the DNA origami tube was not damaged by the prolonged irradiation and by the ROS produced by the photosensitizers (see Figure S13B in the Supporting Information).

**Figure 5:**
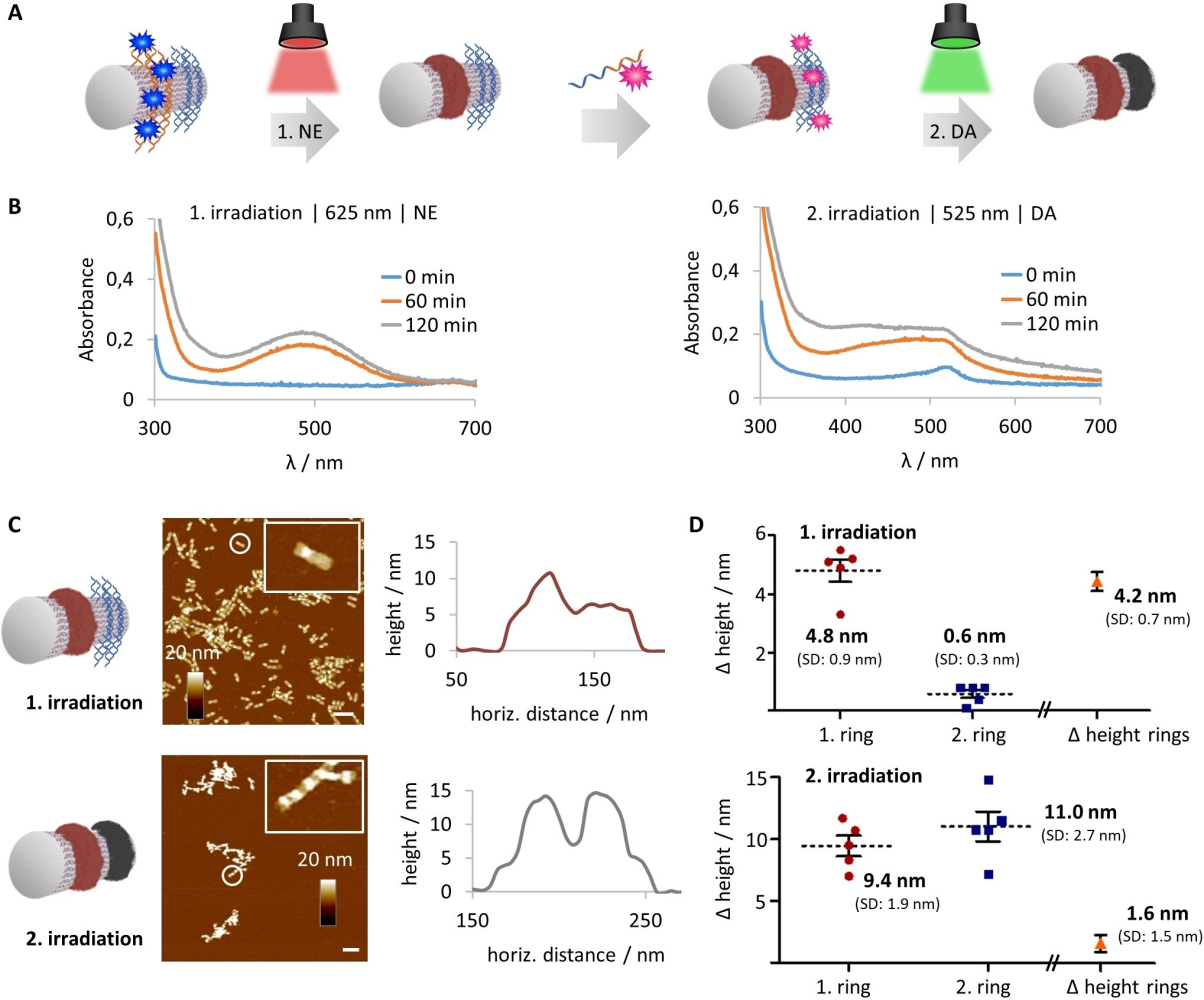
In an advanced two‐step polymerization approach, DNA origami tubes were equipped with two different photosensitizers and incubated with two different monomers, which induced polymer formation at distinct rings at different wavelengths. A) DNA origami tubes were endowed with one ring of G4‐sequences (orange) and one ring of sticky sequences (blue). MB was loaded onto the G4 sequences, NE was added and incubated at 625 nm. After the first irradiation phase, NE was removed by spin filtration. EY‐G4 was annealed on the origami tube, and pDA formation was triggered at 525 nm. B) UV/Vis spectra give the characteristic profiles for pNE and pDA formation, respectively. C) AFM imaging of origami tubes after each step was performed to track and compare the formation of the first and the second polymer ring. Scale bars are 200 nm. Representative height profiles of one ring and two ring structures are depicted. D) Histograms show that only one polymer ring is formed in the first step, whereas a second ring is grown in the second step. Both rings have similar heights (*n*=5; error bars are SEM).

Subsequently, to broaden the approach, we decoupled the loading of both EY and MB photosensitizers into sequential steps to show that DNA hybridization in a post‐polymerization fashion is robust and reliable (Figure 5). The first step involved the attachment of MB onto a DNA origami tube equipped with a single ring of G4 sequences. Irradiation at 625 nm for 2 h with NE (10 mm, pH 6.5) formed the first polymer ring, which could be visualized by AFM and UV/Vis spectroscopy (Figure 5B,C). Excess NE was removed by spin filtration and the second photosensitizer, EY‐G4, was hybridized onto an opposing ring pattern of the same DNA tube using a temperature ramp. The second polymerization step was conducted at 525 nm for 2 h with DA (10 mm, pH 6.5) as the monomer to afford the final nanostructure where pNE and pDA each occupies a single ring. The oxidation profiles of both NE and DA in this dual component system showed consistent polymerization kinetics when compared to the single component system. Furthermore, by comparing the average polymer heights of each irradiation period, sequential ring formation can be tracked (Figure 5D). In the first step, only one position on the origami tube showed polymer formation (4.8±0.9 nm), which is also clearly depicted in the height difference of this polymer ring and the adjacent ring pattern (Δ of 4.2±0.7 nm). In the second step, a second polymer ring was grown at the designated position with a thickness reaching dimensions of the previously grown ring. Interestingly, both polymers exhibit similar heights (Δ of 1.6±1.5 nm).

It is important to note that the existing ring functions as an additional nucleating center, such that activated species from the adjacent ring can diffuse to it, thus resulting in a corresponding increase in height during the activation of the second ring. The presence of all reactive components, namely, monomers, patterned photosensitizers on the DNA origami, and the light source, is essential for the formation of the nanostructure. Control experiments involving only monomer without irradiation, or without embedded photosensitizer did not show polymerization (see Figure S14 in the Supporting Information). In both the coupled and decoupled methods, we demonstrate that sophisticated and multicomponent 3D DNA‐polymer hybrids can be accessed easily.

Additionally, the formation of these polymer patterns can be used as a tool to customize the surface chemistry of the DNA origami. Fundamentally, DNA origami structures are highly anionic due to the polyphosphate backbone and thus require divalent cations for stabilization.^[17]^ Under physiological conditions, DNA origami is prone to degradation through nucleases and the lower concentration of divalent cations in biological fluids.^[18]^ Furthermore, the DNA superstructure itself also has a major impact on the stability of the objects under these conditions.^[19]^ 3D assemblies, for example, significantly slow down the nuclease digestion rate when compared to 2D counterparts.^[20]^

We therefore subjected our DNA structures, bare and polymer‐ringed origami, to cell medium conditions that are encountered when performing cell‐uptake studies. Comparatively, naked DNA structures exhibit a higher level of fragmentation than pNE‐origami when incubated at 37 °C for 24 h in cell medium (see Figure S15 in the Supporting Information).

**Figure 6:**
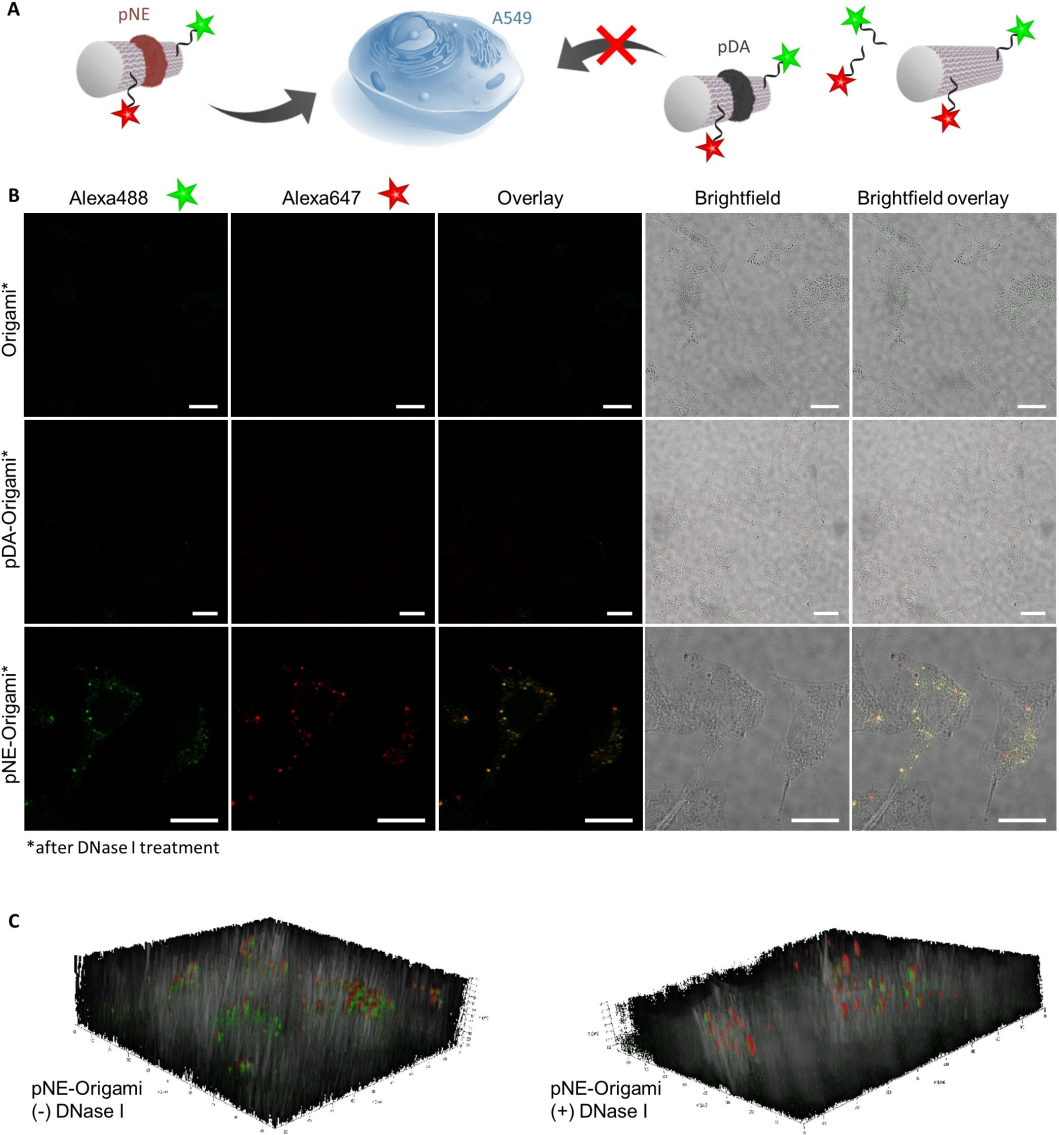
Cellular colocalization of origami nanostructures. A) Schematic representation of the Alexa488‐ and Alexa647‐modification of bare DNA origami tubes as well as pNE‐ and pDA‐ ringed tubes. B) Confocal laser scanning micrographs of A549 cells incubated with Alexa488 and Alexa647 double‐labeled DNA origami samples for 24 h, recorded after DNase I treatment. Scale bars are 20 μm. C) Z‐stacks of pNE‐origami incubated cells without and with DNase I treatment prior to imaging. Control images for buffer only and Alexa‐oligonucleotides are shown in the Supporting Information (see Figure S18A).

Besides stability under physiological conditions, the polyanionic character of DNA prevents its cellular uptake due to repulsive forces towards the negatively charged cellular membrane. Existing strategies to address this challenge include the attachment of targeting functions (i.e. peptides, proteins, and aptamers) that promote receptor‐mediated endocytosis.^[21]^ In contrast, we hypothesized that the coverage provided by the polymer patterns would reduce the effective charge repulsion and thereby improve transport across the cellular membrane. The respective ring‐patterned DNA origami nanostructures with either pNE or pDA were synthesized and annealed with Alexa‐647® oligonucleotide (see Figure S16 in the Supporting Information). At 10 nm, an efficient cellular uptake into A549 lung adenocarcinoma cells by pNE‐origami was observed by confocal laser scanning microscopy after 24 h incubation (see Figure S17 in the Supporting Information). However, significant aggregation was detected for pDA‐origami due to the well‐known adhesiveness of pDA. In agreement with the literature, Alexa‐647®‐labeled DNA origami alone do not show uptake into cells and neither does the Alexa‐647® oligonucleotide. To further characterize the stability of the internalized origami samples, another fluorophore, Alexa‐488®, was annealed onto the opposite end of the tube to facilitate co‐localization studies (see scheme in Figure 6A). The dual‐labeled origami was characterized by AGE followed by gel excision (see Figure S19 in the Supporting Information). By overlaying both channels, colocalization of the two fluorophores indicated that a major proportion of the pNE‐origami structures remain intact upon internalization (see Figure S18 in the Supporting Information). Similarly, controls with bare origami showed no cellular uptake, while the aggregation behavior of pDA‐origami was apparent. Even though the fluorescence colocalization of pNE‐origami samples was positive and the controls excluded the possibility that fragmented components were being taken up, it has to be verified that material is indeed inside the cells. Several reports have shown that the detected fluorescent signal might come from surface‐attached or degraded material only.[^21c^, ^22^] We therefore treated origami‐incubated cells with DNase I to digest cell membrane artefacts. In the case of pDA‐origami, the aggregates were removed with DNase I treatment, thus confirming that the fluorescence signals were largely from membrane‐bound material (Figure 6B, see also Figures S18, S20 in the Supporting Information). In the case of pNE‐origami, although an observable reduction in fluorescence intensity was also observed,^[21c]^ *z*‐stack analysis demonstrated that the pNE‐origami were successfully internalized (Figure 6B,C). The experiment demonstrated that the adhesive forces of pDA were dominant and that the aggregation behavior prevented the uptake of the conjugates into cells. The collective observations indicate that the existence of the pNE layer mediates the repulsive interactions of DNA origami and facilitated uptake into cells. Through these cell experiments, we demonstrate that polymer patterns on DNA origami could be leveraged to alter and modulate interactions at the cellular interface and to enable the uptake of DNA objects.

## Conclusion

In summary, we have explored the patterned and layered growth of different polymers (pNE and pDA) on DNA origami using multiple wavelengths of light. The concept is facilitated by manipulating the interaction of G4s with different photosensitizers (PPIX, EY, and MB) such that their position on the DNA origami can be precisely located. As a consequence, the activity of each photocatalyst can be switched from active to dormant states, and vice versa. Moreover, the fabrication method is flexible so that the sequence of the photopolymerization reaction and/or annealing steps can be changed easily without affecting their efficiencies. The extent of polymer formation can be tracked easily by UV/Vis spectroscopy and AFM imaging, which facilitates structural customization in the *z*‐direction. Furthermore, the polymer patterns altered the intrinsic polyanionic character of the DNA origami while preserving their integrity. By modulating the repulsive forces against the cellular membrane, these hybrid objects could be used for biological applications. In combination, this platform has provided a valuable tool to construct complex polymer‐origami architectures that enable the study of customized surface patterns in nanoscience and biomedicine.

## Conflict of interest

The authors declare no conflict of interest.

## Supporting information

As a service to our authors and readers, this journal provides supporting information supplied by the authors. Such materials are peer reviewed and may be re‐organized for online delivery, but are not copy‐edited or typeset. Technical support issues arising from supporting information (other than missing files) should be addressed to the authors.

Supporting Information
