# Supplementary material for: Multiple Wavelength Photopolymerization of Stable Poly(Catecholamines)‐DNA Origami Nanostructures
Source: Angew Chem Int Ed Engl. 2022 Jan 3;61(8):e202111226. doi: 10.1002/anie.202111226 (PMC9303804; doi:10.1002/anie.202111226)
Supplement: Supplementary file 1 — Supporting Information [file ANIE-61-0-s001.pdf]

Angewandte Chemie
Eine Zeitschrift der Gesellschaft Deutscher Chemiker

Supporting Information

Multiple Wavelength Photopolymerization of Stable Poly(Catecholamines)-DNA Origami Nanostructures

P. Winterwerber, C. J. Whitfield, D. Y. W. Ng, T. Weil**

SUPPORTING INFORMATION

Table of Contents

Experimental Procedures **p. 2**

Materials and Instruments
DNA origami nanostructures
Standard photopolymerization on DNA origami tubes
Two-step photopolymerization on DNA origami tubes
Reactive Oxygen Species Assay (ROS Assay)
Cell culture
Cell experiments
Cell uptake
Cell colocalization
Cell medium stability assay
DNase I digestion assay
Confocal laser scanning microscopy
Atomic Force Microscopy
Agarose gel electrophoresis
DNA extraction from agarose gel
Dynamic light scattering (DLS)

Results and Discussion **p. 4**

SI Figure 1 Photopolymerization of DA with PPIX-G4 at 410 nm and 625 nm.
SI Figure 2 Proposed mechanism for pDA and pNE formation.
SI Figure 3 Reactive oxygen assay (ROS) assay of PPIX, MB, and EY.
SI Figure 4 Photopolymerization of DA with EY-G4 at 525 nm and MB-G4 at 625 nm.
SI Figure 5 DNA origami tube with a diagonal G4 pattern.
SI Figure 6 Aggregation of pDA-ringed DNA origami tubes.
SI Figure 7 Photopolymerization of NE with PPIX-G4 at 410 nm, EY-G4 at 525 nm, and MB-G4 at 625 nm.
SI Figure 8 Dynamic light scattering analysis of bare and pNE-origami for 3 days.
SI Figure 9 Agarose gel electrophoresis of DNA origami tubes before and after polymerization.
SI Figure 10 Stepwise layer-by-layer polymerization of NE and DA.
SI Figure 11 Control experiments for layer-by-layer polymerization.
SI Figure 12 Activity and dormancy of EY and MB at opposite wavelengths.
SI Figure 13 Ring-by-ring polymerization of pDA.
SI Figure 14 Control experiments for ring-by-ring polymerization.
SI Figure 15 Cell medium stability assay of bare and polymer-ringed origami tubes.
SI Figure 16 Fluorescence calibration curve for cell uptake studies.
SI Figure 17 Cellular uptake of DNA origami structures labelled with Alexa647.
SI Figure 18 Co-localization cell studies and DNase I assay.
SI Figure 19 Characterization of dual labelled origami structures for cell uptake studies.
SI Figure 21 Confocal z-stack analysis of pNE-origami cell uptake studies.

Appendix **p. 18**

DNA sequences and patterns

References **p. 22**

Experimental Procedures

Materials and instruments
All solvents and chemicals were purchased from commercial sources and were used without further purification. DNA oligonucleotides (staple strands, sticky strands, G4 staple strands, G4-complementary strands, and folding strands) were purchased from Sigma-Aldrich. M13mp18 plasmid DNA was purchased from tilibit nanosystems. Annealing of DNA origami structures and hybridizing of sticky sequences was performed on a Bio-Rad MyCycler^{TM} Thermal Cycler. UV/Vis and fluorescence spectroscopy were conducted on a Spark® Multimode Microplate Reader by Tecan. Buffers were adjusted on a Mettler Toledo SevenExcellence^{TM} equipped with the InLab Nano ph electrode. Photopolymerizations were conducted in a house-built photobox that is equipped with interchangeable LED-arrays (96 LEDs) of 410 nm, 525 nm, and 625 nm.

DNA origami nanostructures
DNA origami tubes were prepared by mixing M13mp18 Scaffold DNA (1 equiv.), staple strands (8 equiv.), G4 staple strands (8 equiv.), stickyA strands (8 equiv., if necessary), and folding strands (16 equiv.) in origami buffer (1 mM Na_2EDTA, 5 mM NaCl, 5 mM TRIS, 12 mM $MgCl_2$, pH 8). Annealing was performed by running a program from 70 °C to 20 °C over 2 h (0.5 °C/min to 35 °C, 1 °C/min to 20 °C) and the obtained DNA origami structures were purified by PEG precipitation.[1] Therefore, the PEG solution (15% PEG_{8000} (w/v), 5 mM TRIS, 1 mM Na_2EDTA, 505 mM NaCl) was added to the reaction solution at a volume ratio of 1:1 and centrifuged for 25 min at 12.5 rpm, room temperature (RT). The supernatant was removed, and the resuspended pellet was precipitated by applying the PEG precipitation method for additional two times. Sample concentration was determined by Spark® 20M with Nanoquant plate^{TM}. DNA origami structures were stored in origami buffer at 4 °C.

Standard photopolymerization on DNA origami tubes
Standard photopolymerization on DNA origami tubes (44x G4 catalytic centers per tube) was carried out in a total reaction volume of 50 µL in a UV-star 384 well plate. Stock solutions of compounds were prepared in reaction buffer (10 mM BIS-TRIS, pH 6.5) at the following concentrations: photosensitizers (PS) at 10 µM; monomers at 0.5 M. A typical reaction was conducted as follows: DNA origami with G4-sequences (10 nM, final concentration) was incubated with PS (1.5 equiv., relative to the amount of G4-sequences) in reaction buffer for 30–60 min. Monomer (10 mM, final concentration) was added and the plate was placed in a house-built photobox. After a predetermined irradiation time interval, polymerization was stopped by switching off the light source and polymer-DNA objects were purified using size exclusion chromatography (200 µL Sephacryl S-400 HR, equilibrated with reaction buffer; centrifuge settings: 2 min, 0.8 g, RT) or 100K spin filtration (5 g, 10 min, 2 times; recovered at 1 g, 2 min). For purification steps, a mixture of origami buffer and reaction buffer ratio (1:4) was utilized.

Two-step photopolymerization on DNA origami tubes
Layer-by-layer polymerization: DNA origami tubes were equipped with one ring of G4 sequences (44 sequences) and incubated with MB under standard conditions (see above) for 30 min. NE was added and the reaction solution was subjected to standard polymerization conditions at 625 nm for 2 h. After 100K spin filtering, DA was added and irradiated for 2 h at 625 nm. Polymer-ringed origami tubes were recovered by 100K spin filtering and stored at 4 °C.
Ring-by-ring polymerizations: DNA origami tubes were equipped with one ring of G4 sequences and on ring of stickyA sequences (44 sequences each). Methylene blue was incubated with G4 sequences on origami tubes in an 1.5 molar excess (related to the amount of G4 sequences) for 30 min. Eosin Y was preloaded into G4 complementary strands in an 1.5 molar excess for 30 min and either annealed with MB-loaded origami tubes before photopolymerization or after the first irradiation phase. Hybridizing of EY-G4 strands (1.5 equiv. compared to stickyA sequences on DNA origami tubes) was performed by a temperature ramp from 35 °C to 20 ° in 5 °C steps (holding each step for 15–30 min). After annealing, excess staple strands were removed by 100K spin filtering. For continuous photopolymerization, MB- and EY-loaded tubes were incubated with DA (standard polymerization conditions, see above) and irradiated at 625 nm and 525 nm for 3 h each. For decoupled photopolymerization, MB-loaded tubes were incubated with NE and subjected to standard photopolymerization at 625 nm for 2 h. After purification (100K spin filtering) and annealing with EY-G4 strands, DA was added, and polymerization continued at 525 nm for 2 h. In both approaches, polymer-ringed origami tubes were purified by 100K spin filtering and stored at 4 °C.

Reactive Oxygen Species Assay (ROS Assay)
Reactive oxygen species assay of photosensitizers (PS) was conducted in a total reaction volume of 150 µL in a 96 well plate. Wells were charged with PS (6.6 µM), *N*,*N*-dimethyl-4-nitrosoaniline RNO (70.7 µM) and imidazole (7.5 mM) in BIS-TRIS buffer (pH 6.5) and exposed to the respective wavelength or kept in dark. Absorbance scans from 300–500 nm were conducted at several time points within 30 min. The decrease in absorbance of RNO could be followed at 438 nm.

Cell culture
A549 cells were cultured at 37 °C and 5% CO2 in Dulbecco's Modified Eagle's Medium (DMEM, high glucose), which was supplemented with 10% FBS, 1% penicillin/streptomycin and 1× Minimum Essential Medium (MEM) non-essential amino acids.

Cell experiments
A549 cells were cultured in DMEM medium containing 10% fetal bovine serum and 1% penicillin/streptomycin as well as 1% MEM non-essential amino acids. Cells were seeded into confocal well plates and left for 24h to adhere at 37 °C, 5% CO_2.

Cell uptake
For cell uptake studies, DNA origami tubes with a ring of either pDA or pNE were synthesized (according to standard polymerization protocol, MB, 625 nm, 3 h). Furthermore, origami tubes were equipped with 22 sticky sequences for Alexa-647® oligonucleotide labelling ("sticky C"). Dye-labelling was conducted in a 10-fold molar excess by a 1 hour temperature ramp (from 35 °C to 20 ° in 5 °C steps), followed by 100K spin filter purification. Control samples without polymer coating were labelled in the same way.
Cells were seeded at a density of 5,000 cells/well in an 10-well confocal well plate. After adhering for 24 h, cells were treated with the Alexa647-labeled sample for 24h at 37 °C. Sample solutions were diluted to 20 nM (Alexa647 concentration via a standard curve) in

cell media for incubation with the cells. After the incubation time, the media/sample solution was replaced with fresh media. Cells were then imaged by confocal laser scanning microscopy (SI Figure 12).

Cell colocalization

Besides the polymer ring, DNA origami tubes were additionally equipped with 22 sticky sequences each for Alexa-647® and Alexa-488® oligonucleotide labelling (standard protocol, see above; "stickyC for Alexa-647® and stickyA for Alexa-488®). Control samples without polymer coating were labelled in the same way.

Cells were seeded at a density of 5,000 cells/well in an 10-well confocal well plate. After adhering for 24 h, cells were treated with the double-labeled sample for 24 h at 37 °C. Sample solutions were diluted to 10 nM (Alexa647 concentration via a standard curve) in cell media and incubated with the cells. After the incubation time, the sample solution was replaced with fresh media. Cells were then imaged by confocal laser scanning microscopy (Figure 4 and SI Figure 13).

Cell medium stability assay

To test the stability of the employed DNA origami structures under cell medium conditions, samples were incubated with cell medium in an 1:1 ratio (approx. 50 fmol, total volume of 10 or 20 µL) for 24 h at 37 °C in a thermocyler with a heated lid. After incubation time, total sample volume was loaded on gel for AGE. For comparison, an analogue series of samples was mixed with cell medium right before loading onto the gel.

DNase I digestion assay

To digest DNA origami tubes that were only attached to the cell's exterior after overnight incubation, DNase I (20 U) was added to the samples and incubated for 1 hour at 37 °C prior to imaging.

Confocal Laser Scanning Microscopy

Cells were imaged on a Leica TCS SP5 and a Visitron Spinning Disc microscope with an argon laser for excitation at 488 nm for Alexa488 (emission 498-540 nm), and a HeNe laser for excitation at 633 nm for Alexa647 (emission 657-757 nm). Z-stack images was acquired using a Leica Stellaris 8 confocal microscope equipped with a white light laser with tunable excitation wavelengths between 440 – 790 nm. Alexa 488 is excited at 488 nm and emission was collected using the HyD® X detector at 498-540 nm. Alexa 647 is excited at 647 nm and emission was collected using the HyD® R detector at 657-757 nm.

Atomic Force Microscopy (AFM)

Atomic force microscopy was performed in liquid state with a Bruker Dimension FastScan Bio AFM equipped with the ScanAsyst mode. Sample solution (30 µL, 1-2 nM in origami buffer) was added onto a freshly cleaved mica substrate and incubated for 5 min to allow deposition of the origami structures. Remaining solution was removed and 300 µL origami buffer was applied onto the mica surface, forming a droplet for measuring in liquid. Samples were scanned with scan rates between 1 and 2 Hz. Images were processed with NanoScope Analysis 1.8.

Agarose gel electrophoresis (AGE)

Agarose gel electrophoresis was performed on 1% agarose gels (TBE, stained with EtBr, purchased from Bio-Rad or self-casted with 1 × TBE when no staining was desired), equipped with 8 wells. The gels were run on ReadySub-Cell GT Cells from Bio-Rad using 1 × TBE buffer as the running buffer. DNA Gel Loading dye (6 ×) was used for sample preparation (approximately 50 fmol origami) with a total volume of 6, 12, or 18 µL, depending on sample concentration. "GeneRuler DNA Ladder Mix" (100–10000 bp) was used as for the marker. Electrophoresis was conducted at 90 V for 60–100 minutes at 4 °C. Image was taken with G:BOX Chemi Gel Doc System from Syngene or under UV-excitation with a camera.

DNA extraction from agarose gel

For DNA extraction, TBE gels were casted and run as mentioned above. Areas below the wells were defined and cut according to the band pattern found under UV irradiation. The excised gel piece was placed into a Costar® Spin-X® Centrifuge Tube Filter (0.45 µm pore NY membrane), left at – 20°C for 1 hour and subsequently centrifuged at 10 g for 10 minutes. 100 µL of the filtrate was placed into a black 384 well plate and subjected to fluorescence measurements.

Dynamic light scattering (DLS)

DLS measurements were performed at 25 °C using a Malvern ZetaSizer Nano S from Malvern Instruments Ltd. with a He/Ne Laser (λ = 633 nm) at a fixed scattering angle of 173°. All measurements were performed in triplicate. The obtained data was processed by cumulant fitting for Dz and PDI, or by CONTIN fitting for intensity-weighted particle size distribution. For DLS, triple amount of DNA origami (compared to standard polymerization) was photopolymerized (MB, 625 nm), purified and brought to 150 µL with reaction buffer. As a control, 10 µL bare origami tubes (50 nM) were diluted to 150 µL with reaction buffer. Samples were filtered prior to measurement through PFTE (hydrophilic) syringe filters (0.45 µm pore size).

Results and Discussion

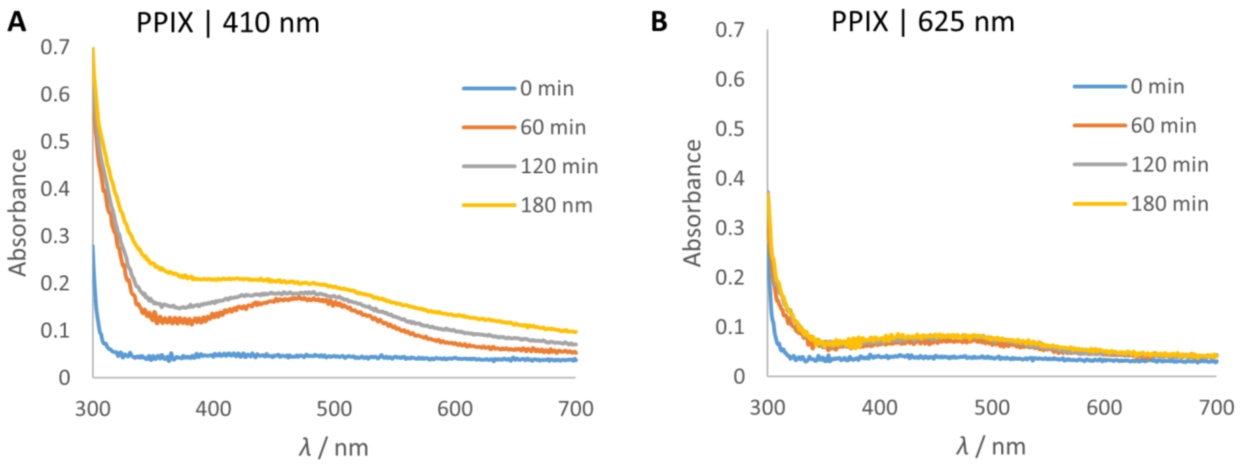

Figure 1 Photopolymerization of dopamine with PPIX-loaded DNA origami tubes under irradiation at 410 nm and 625 nm, respectively. Reaction is followed spectrometrically. (A) Irradiation at 410 nm can successfully induce oxidation and polymerization of dopamine. (B) Irradiation at 625 nm only leads to a reduced generation of oxidized dopamine species.

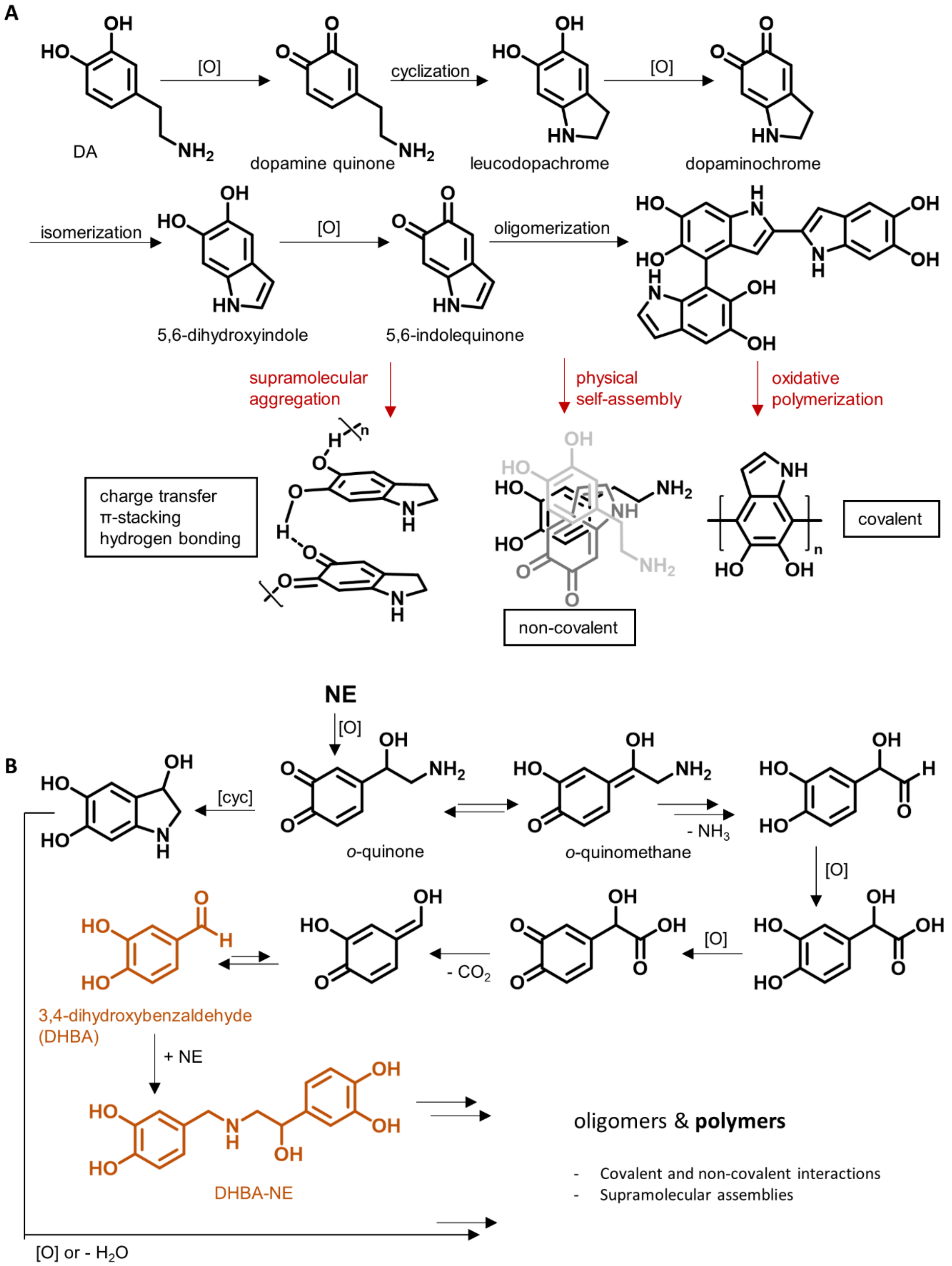

Figure 2 Proposed mechanism for pDA formation[2] (A) and pNE formation[3] (B) according to the literature. Both mechanisms are still under ongoing elucidation. Importantly, the polymeric structures are not only built on covalent bonds, but also non-covalent interactions, supramolecular assemblies, charge transfer and (cation-) π-stacking are present. In contrast to pDA, pNE formation is characterized by the occurrence of 3,4-dihydroxybenzaldehyde (DHBA), that can react with the monomer NE again, forming DHBA-NE. This molecule is said to be responsible for the ultrasmooth surface of pNE.

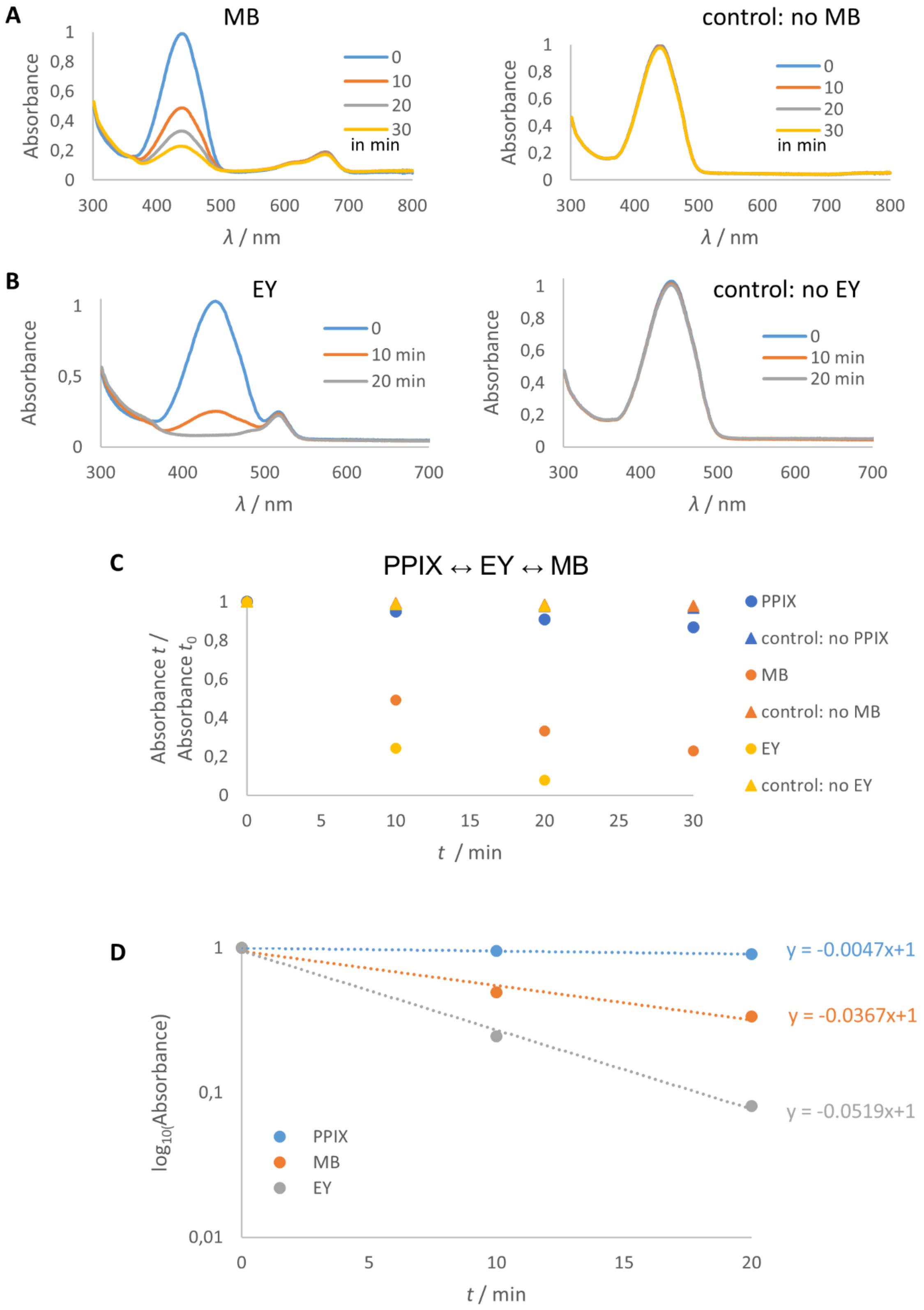

Figure 3 ROS assay of the utilized photosensitizers methylene blue (MB) and eosin y (EY) in the presence of imidazole and p-nitrosodimethylaniline (RNO). Decrease in absorbance of RNO due to singlet oxygen generation can be followed at 438 nm. (A) Methylene blue was irradiated at 625 nm (5 mA), and (B) eosin y was irradiated at 525 nm (5 mA) for the respective timeframes. (C) Relative absorbance decrease at 438 nm shows MB, EY and protoporphyrin IX (PPIX) in comparison. (D)

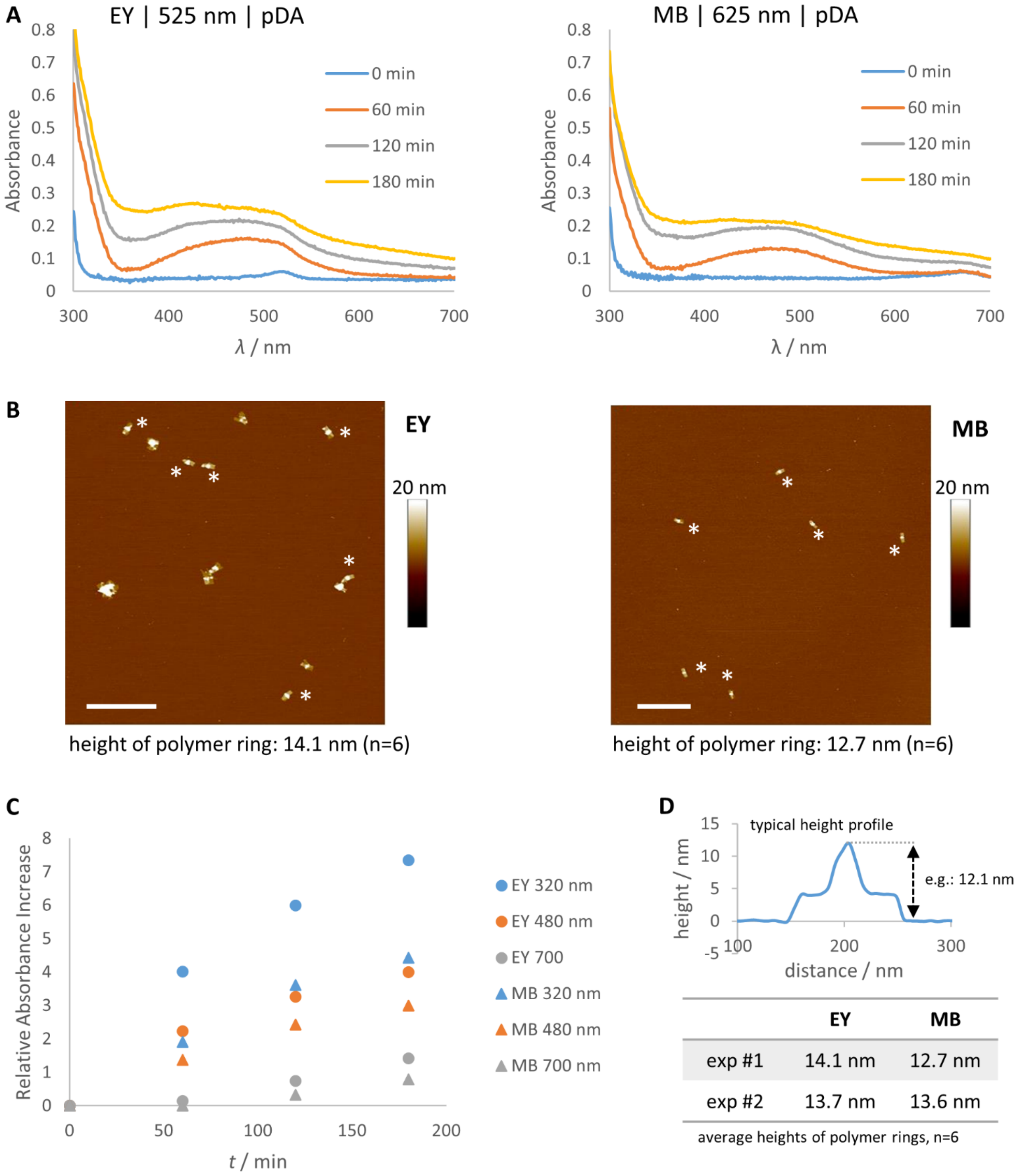

Figure 4 Photopolymerization of dopamine on DNA origami tubes with the photosensitizers eosin Y (EY) and methylene blue (MB) that are irradiated at 525 nm and 625 nm for 3 h, respectively. (A) Successful polymerization in both cases is confirmed by UV/Vis spectroscopy. (B) Topographical AFM images of pDA-ringed origami tubes after photopolymerization with EY (left) and MB (right). The average height of the polymer rings is depicted below the images. Scale bar: 500 nm. (C) Reaction kinetics of EY and MB are compared by plotting relative absorbance increase of the oxidized species dopaminochrome (320 nm), oligomers (480 nm), and polydopamine (700 nm). EY shows higher reaction rates than MB. (D) Statistical overview of the height of the polymer rings when either EY or MB is employed (two experiments each). The typical height image explains how values are taken. Polymer rings are slightly higher when EY is employed but no significant impact on the final product is noted.

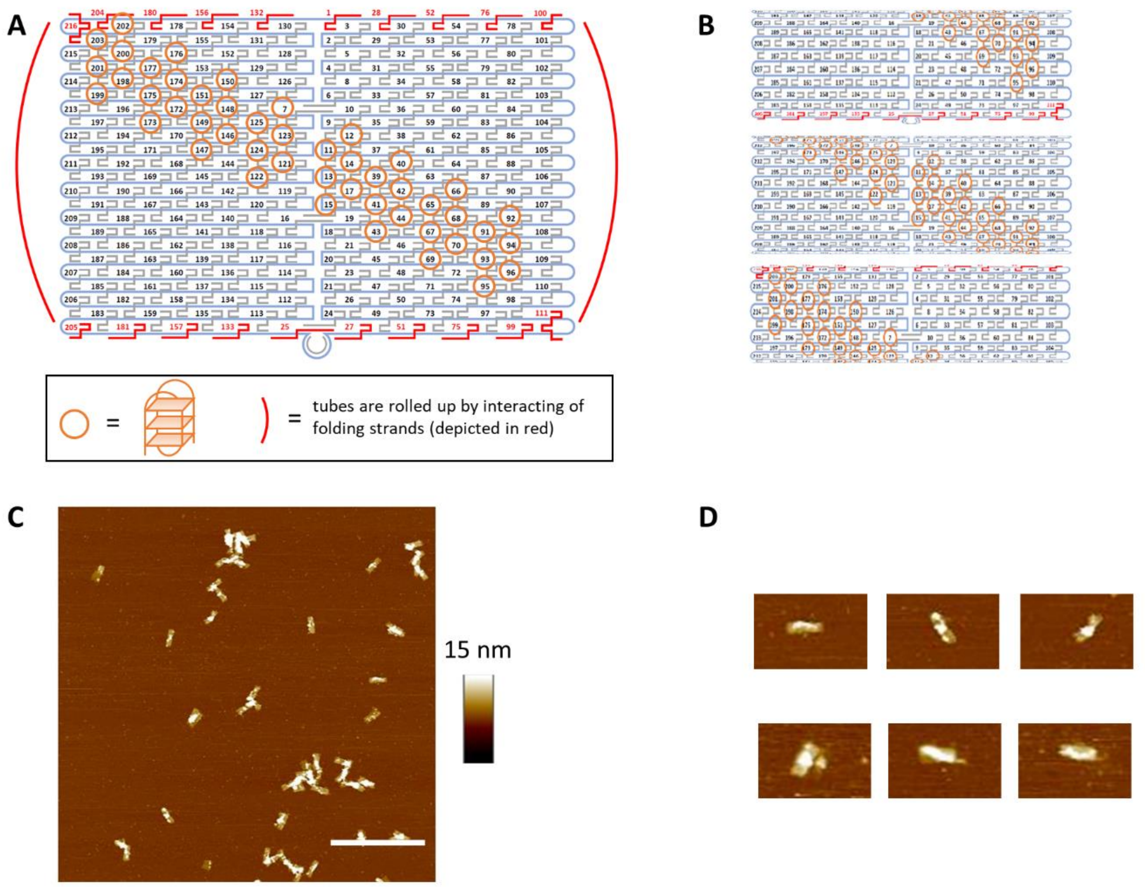

Figure 5 Photopolymerization of dopamine from a DNA origami tube with a diagonal pattern. (A) 48 G4 sequences are arranged in a diagonal pattern on the tube's surface. (B) Here, the top view on the structures deposited on an AFM grid can vary, depending on the orientation. (C) Exemplary overview AFM image of the obtained structures after polymerization. Scale bar: 500 nm. (D) Close-ups of a few representative pDA origamis. The diagonal pattern is only visible in a few cases due to the random orientation of the origami tube. For characterization purposes, ring patterns remain the pattern of choice because of its verticle symmetry and thus removing the orientation factor.

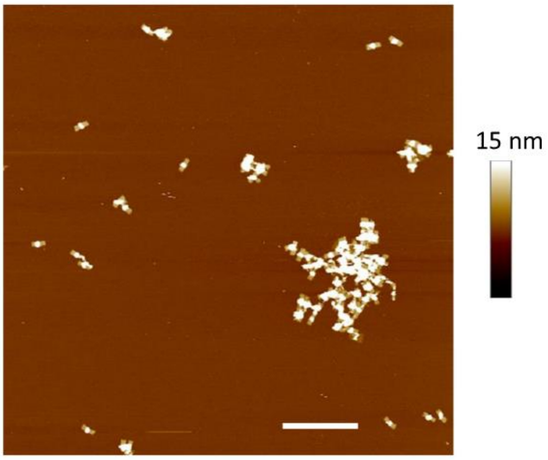

Figure 6 Due to the strong adhesiveness of pDA, polymer-ringed DNA origami tubes tend to aggregate. Scale bar: 500 nm.

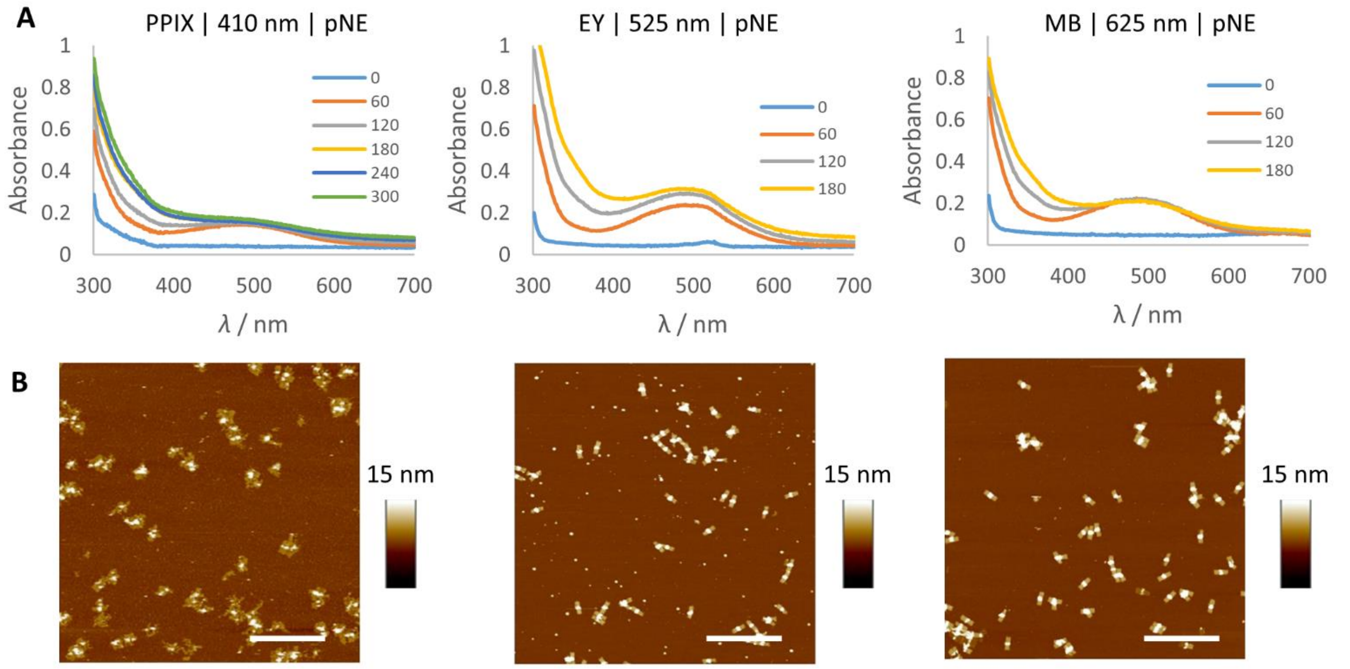

Figure 7 Photopolymerization of norepinephrine (NE) can be stimulated through PPIX, EY, and MB at the respective wavelength. (A) Polymerization can be followed spectrometrically. (B) Topographical AFM images confirm polymer formation at the designated areas. Scale bar: 500 nm.

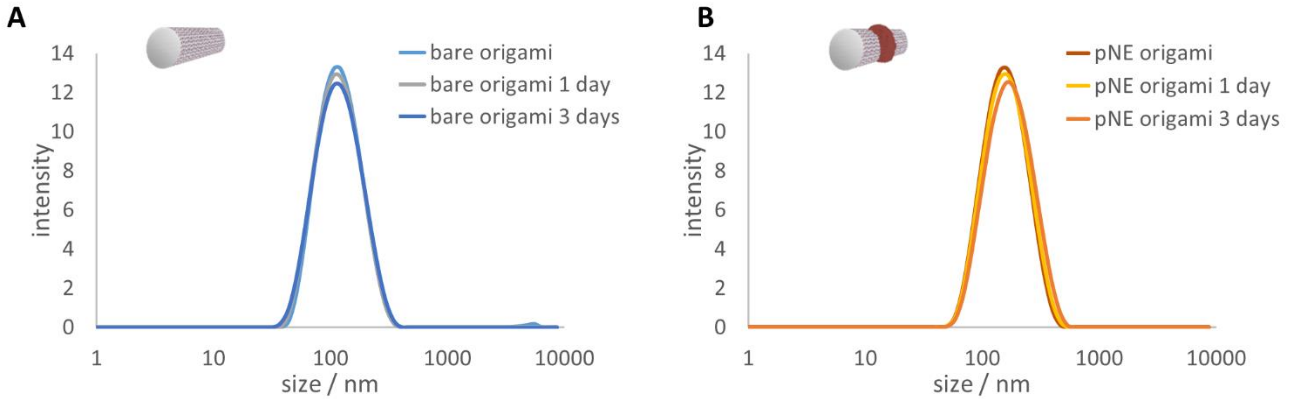

Figure 8 Dynamic light scattering reveals that for both samples, (A) bare origami and (B) pNE-ringed origami tubes, no agglomeration occurs over the course of at least 3 days.

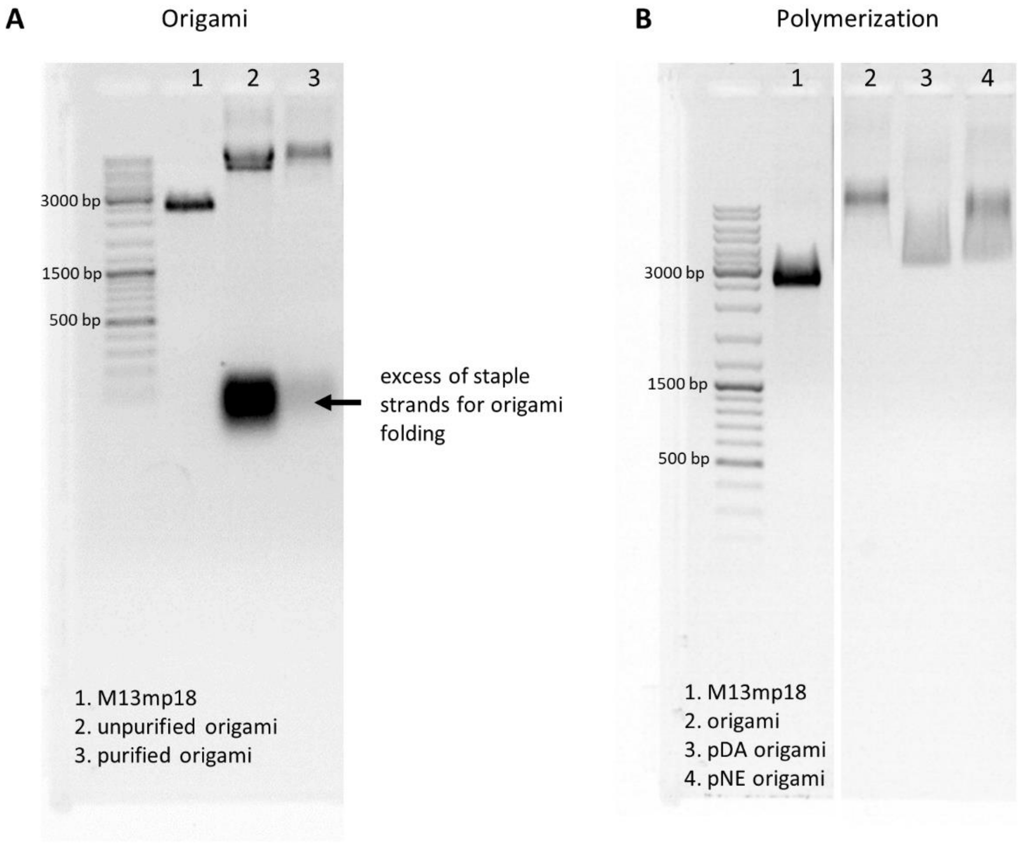

Figure 9 Characterization of (A) DNA origami structures and (B) DNA origami structures before and after pDA and pNE formation by agarose gel electrophoresis. After annealing of scaffold DNA (M13mp18) and an excess of staple strands, the folded DNA origami tubes are apparent as a new band of lower mobility (A2,3); excess of staple strands is removed by purification. Polymer-modification (pDA and pNE) is further visible by a changed running behavior compared to the precursor origami band (Gel B). The polymer bands also show some smearing effect, potentially indicating aggregation of the structures. Especially in the case of pDA-modification, we sometimes also observe the retaining of some material in the well of the gel, also demonstrating the presence of aggregates (gels not shown here). 1 % agarose TBE gels stained with EtBr were used, and run at 90 V for 60 min (Gel A) and 95 min (Gel B) at 4 °C. Please note: Gel (B) is cropped to only show lanes of interest, however, no stretching or shrinking was done, so bands are still comparable.

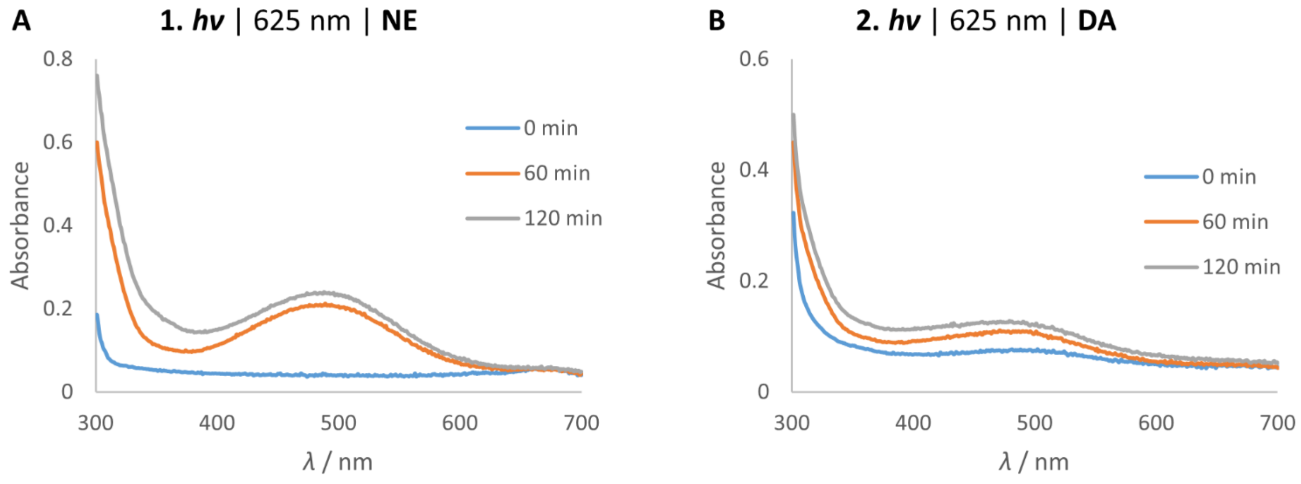

Figure 10 Stepwise layer-by-layer polymerization of the two monomers norepinephrine and dopamine on DNA origami tubes that are activated with MB and irradiated at 625 nm. UV/Vis spectroscopy confirms formation of (A) polynorepinephrine in the first irradiation phase and (B) polydopamine in the second irradiation phase. Spin filtration was performed after step 1 to exchange monomers.

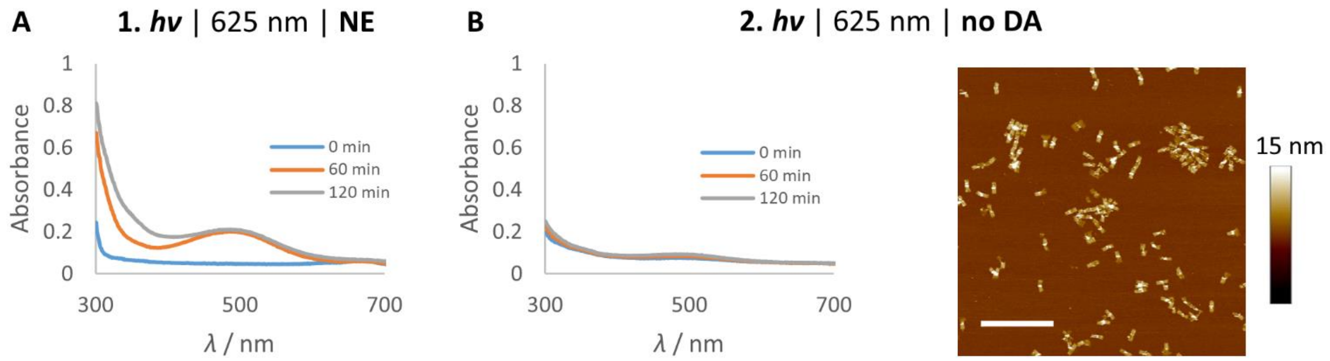

Figure 11 Control experiment for layer-by-layer polymerization, showing that it is in fact pDA that is formed in step 2, not pNE. (A) After pNE is generated in the first irradiation period, NE is removed from the reaction solution and no dopamine is added for the 2nd irradiation phase. (B) UV/Vis spectroscopy shows that no polymer is formed in step 2 and in AFM imaging, only height increase for pNE rings from step 1 is found.

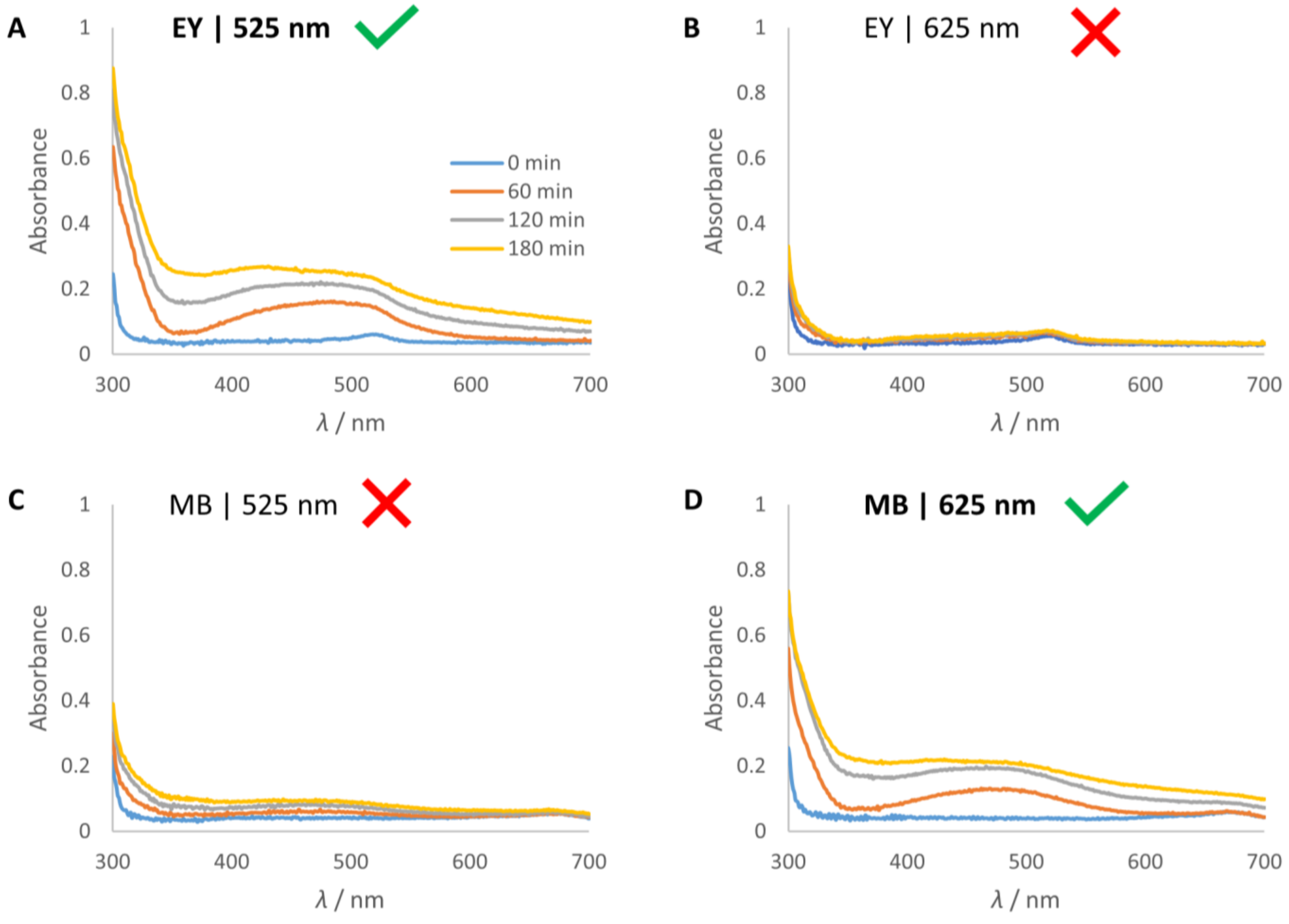

Figure 12 For a two-step polymerization with two photosensitizers, it is important that each photosensitizers only responds to the designated wavelength. This holds true for eosin Y, active at 525 nm (A) and dormant at 625 nm (B), and methylene blue, dormant at 525 nm (C) and active at 625 nm (D), respectively. Polymerization was conducted on standard origami tubes for 3 h with dopamine as the monomer.

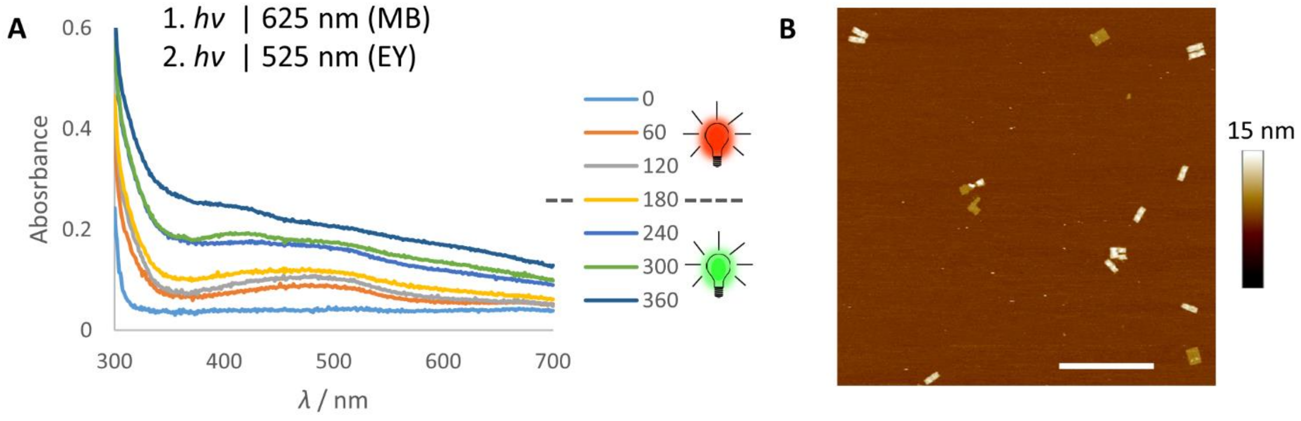

Figure 13 Photopolymerization of dopamine of MB- and EY-loaded origami tubes can be stimulated through irradiation at the respective wavelength (625 nm and 525 nm, respectively). (A) UV/Vis spectroscopy confirms the continuous generation of oxidized species and polymer. (B) AFM images demonstrate the origami's integrity even after irradiation of 6 h in the presence of photosensitizers.

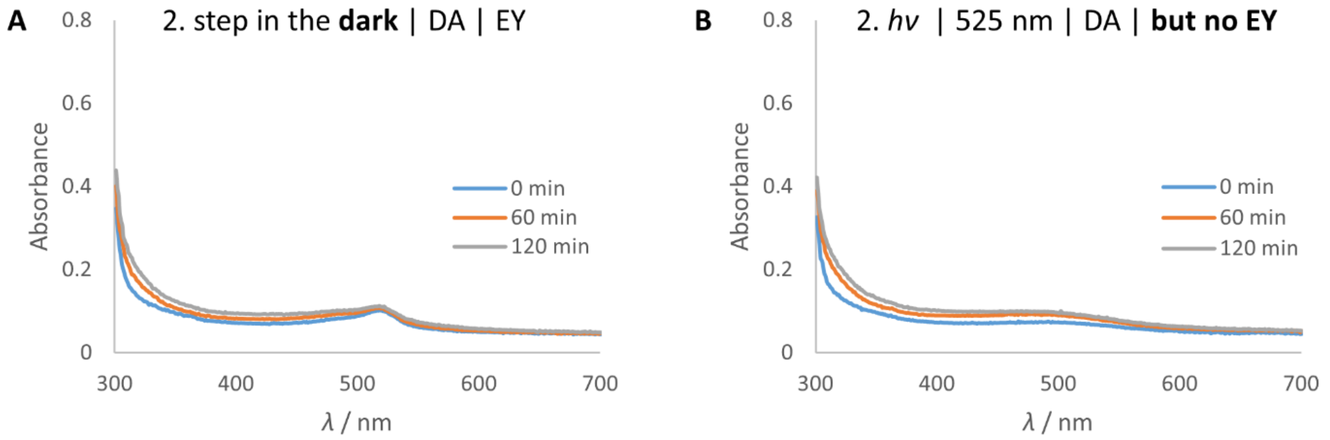

Figure 14 For a sequential two-step polymerization, the presence of all the reactive components is necessary. (A) When photosensitizer (EY) and monomer (DA) are added for step 2, but the reaction is performed in the dark, no polymerization is noted. (B) Same holds true when each component except for the photosensitizer (EY) is applied for the second polymerization step.

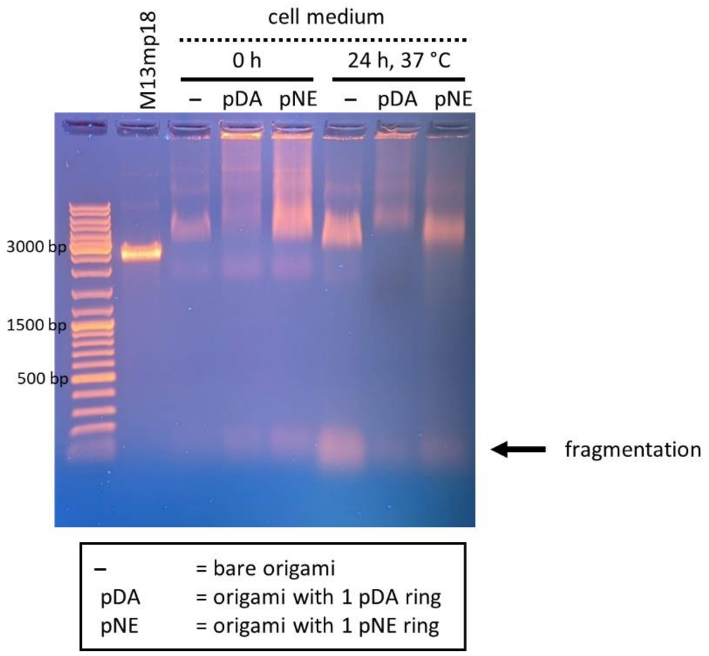

Figure 15 Cell medium stability assay of bare origami tubes as well as polymer-coated ones. Samples were incubated at 37 °C for 24 h and compared to samples that were mixed with cell medium prior to loading the gel. Even the bare origami tubes do show a certain stability under the applied incubation conditions, but also fragmentation. Polymer-ringed origami samples show less fragmentation, especially for pDA, material is stuck in the well, most likely caused by retained aggregates. A 1 % agarose TBE gel stained with EtBr were used, and run at 90 V for 90 min at 4 °C.

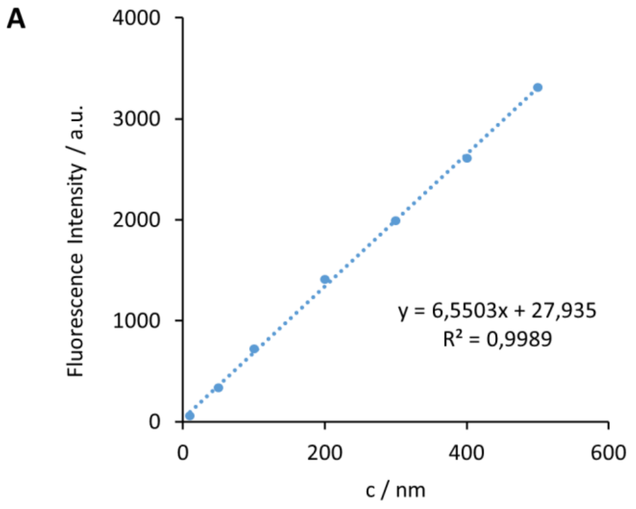

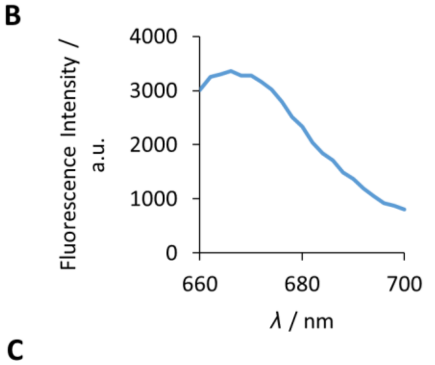

C

	$F_{666\,nm}$	c / nM
pNE origami	478	68.7
bare origami	698	102.3

Figure 16 Fluorescence concentration of the origami samples subjected to cell uptake studies was calibrated by an Alexa647 standard curve. (A) A dilution series of Alexa647-oligonucleotide from 500–10 nM was measured in duplets (excitation at 645 nm, emission scan from 660–700 nm, emission maximum at 666 nm). (B) Representative emission scan for Alexa647-oliognucleotide at 500 nM. (C) Fluorescent signal and concentration of DNA samples was calibrated using the Alexa647 standard curve.

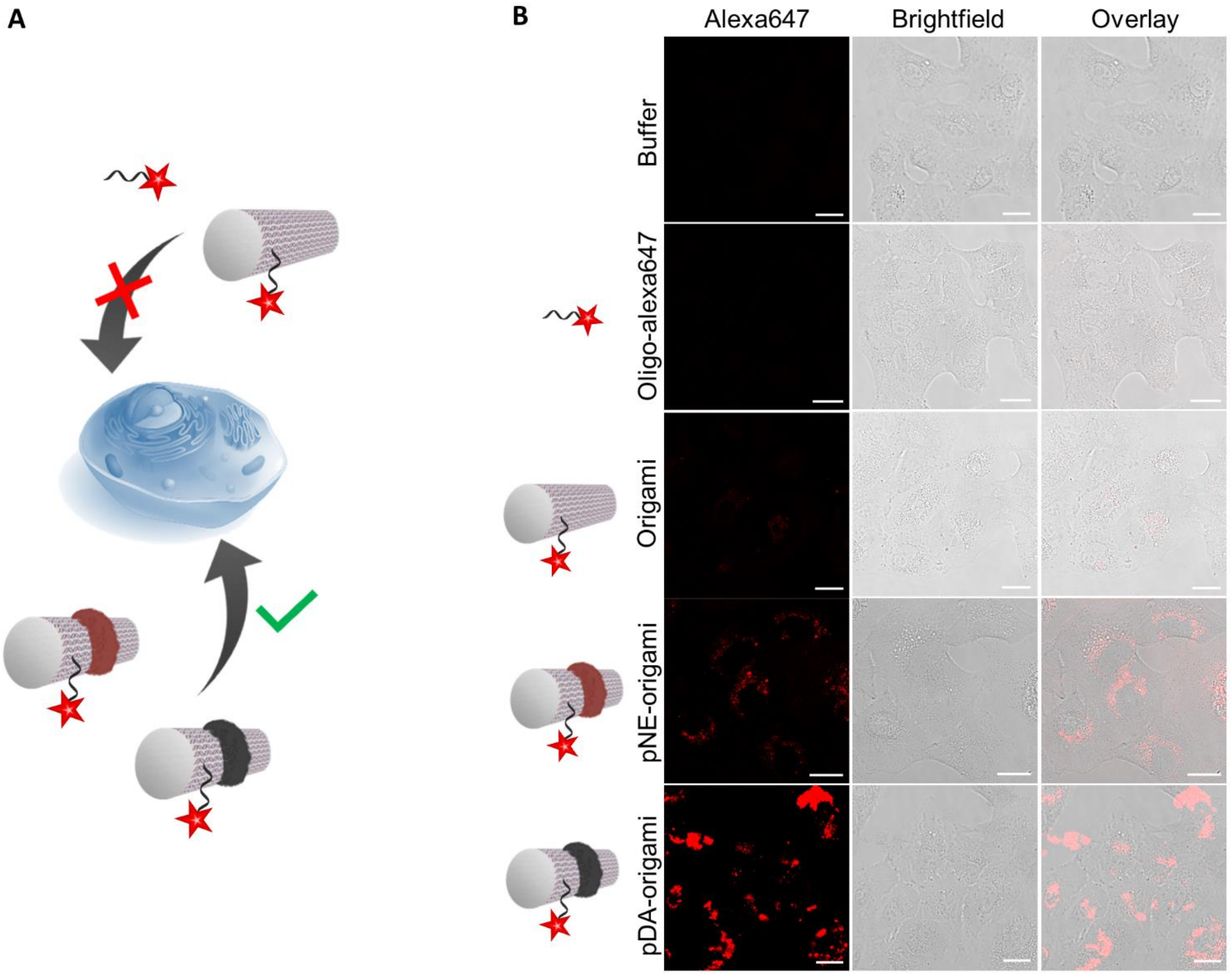

Figure 17 Cellular uptake of origami nanostructures. (A) Free Alexa647-oligonucletoides, bare origami tubes, pNE- and pDA-ringed origami tubes (all origami structures labelled with Alexa647) are incubated with A549 cells. (B) Confocal laser scanning micrographs of A549 cells treated with samples from (A) for 24 h. Scale bar 20 µm.

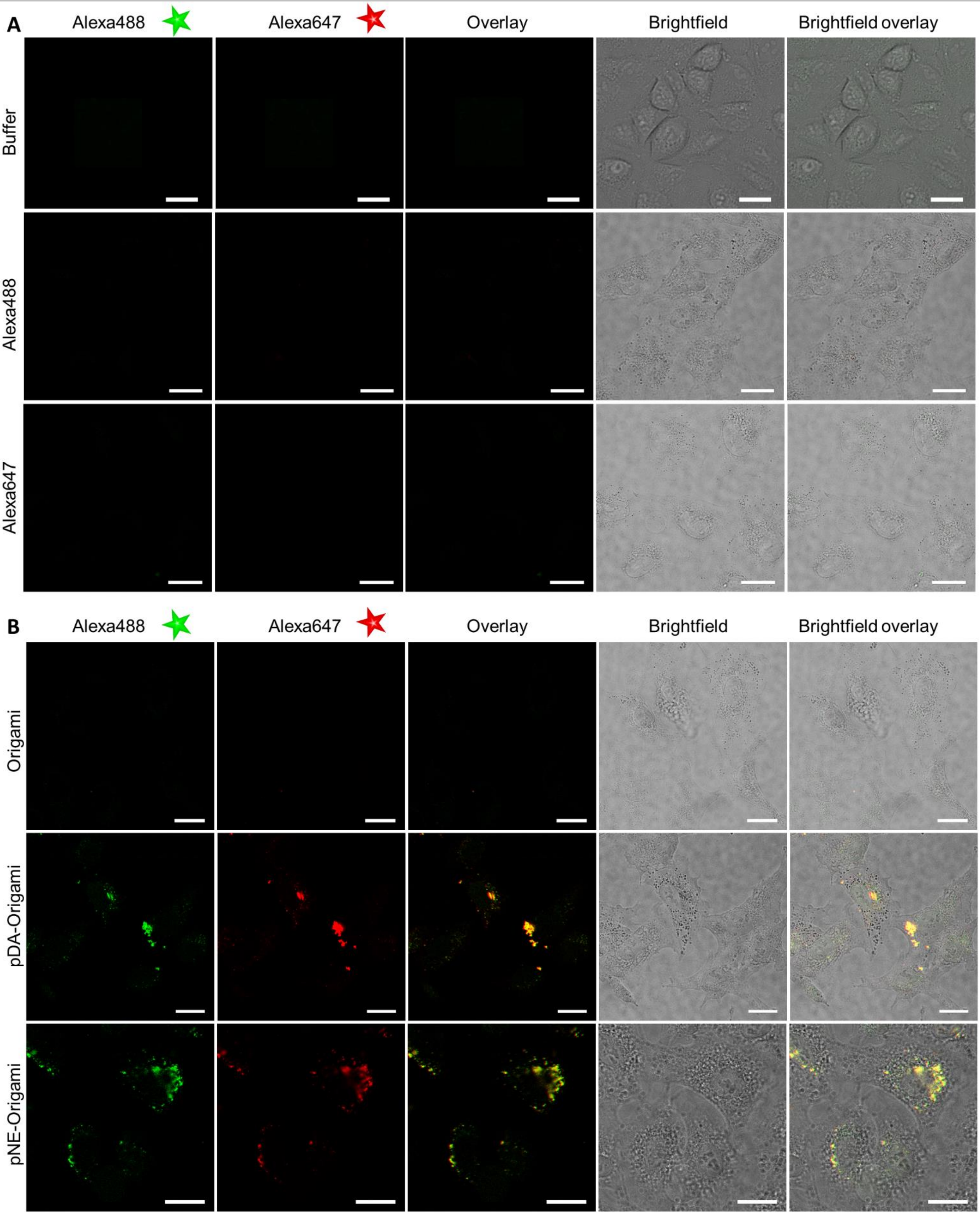

Figure 18 Confocal laser scanning micrographs of A549 cells incubated with (A) buffer only (control), free Alexa488- and Alexa647-oligonucleotides, and (B) double-labeled DNA origami samples (bare origami, pDA-origami, pNE-origami) for 24 h. Scale bars are 20 µm.

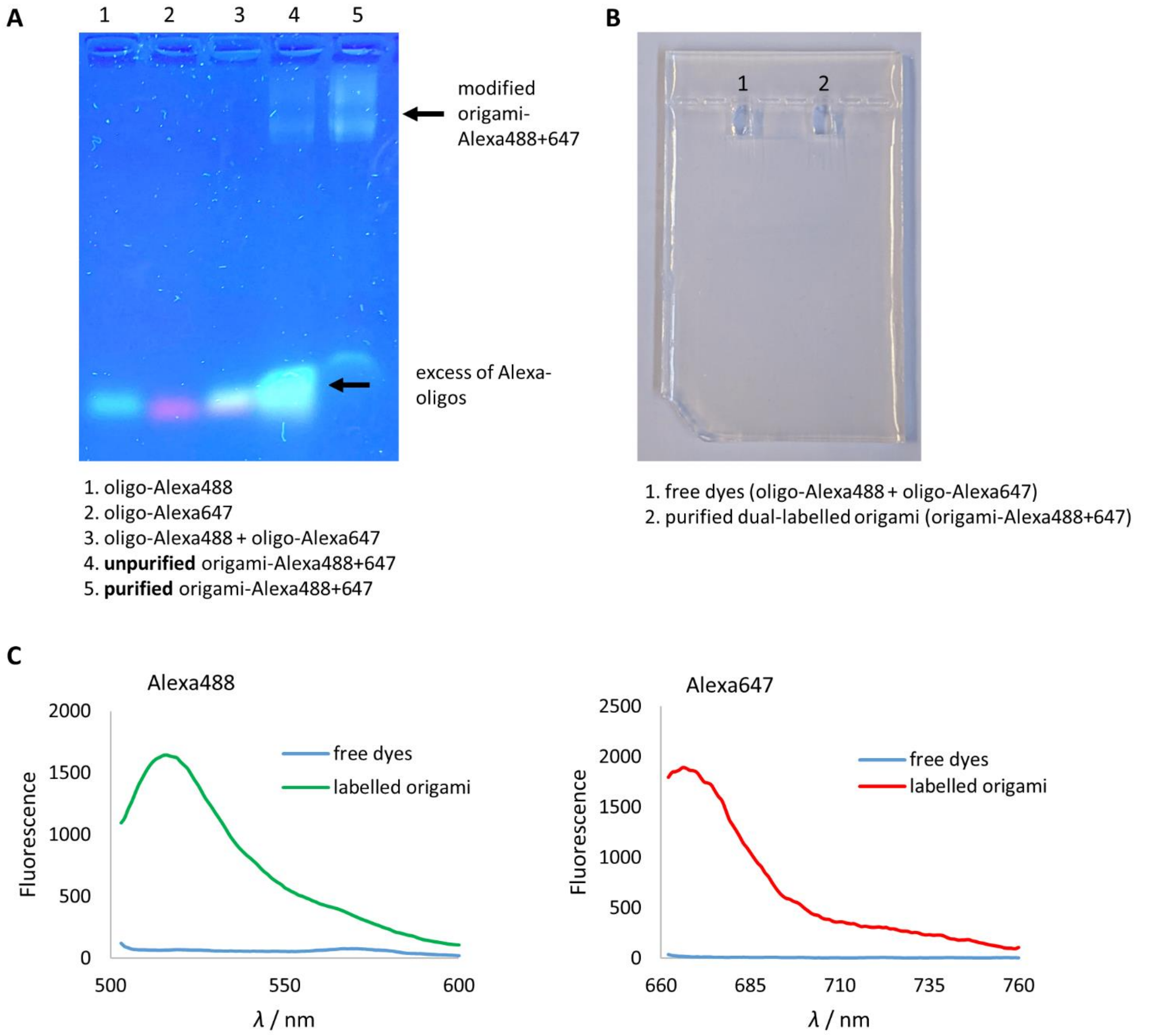

Figure 19 Characterization of dual-labelled origami structures for cell studies by agarose gel electrophoresis and subsequent fluorescence measurements. (A) AGE was performed with the Alexa-oligonucleotides, the unpurified as well as the purified origami sample after modification with both fluorophores. Gels were visualized on a UV plate without further staining. Whereas the free dyes show their respective emission color or a mixture thereof in the lower part of the gel; fluorescent origami structures are visible in the upper part of the gel. Excess of Alexa-oligonucleotides for origami modification was removed by purification. (B) A gel was loaded with both Alexa488- and Alexa647-oligonucleotides (lane 1) and purified, dual-labelled origami sample (lane 2). The areas right below the wells were cut, frozen, centrifuged, and the filtrate was subjected to subsequent fluorescence measurements in a Tecan Plate Reader. (C) Excitation of Alexa488 and Alexa647, respectively, revealed the presence of the Alexa fluorophores in the origami sample but not in the control sample, demonstrating successful labelling with both dyes. Emission spectra of Alexa488 and Alexa647 were recorded from 503–600 nm after excitation at 480 nm and from 662–760 nm after excitation at 640 nm (bandwidths: 10 nm), respectively, and smoothed.

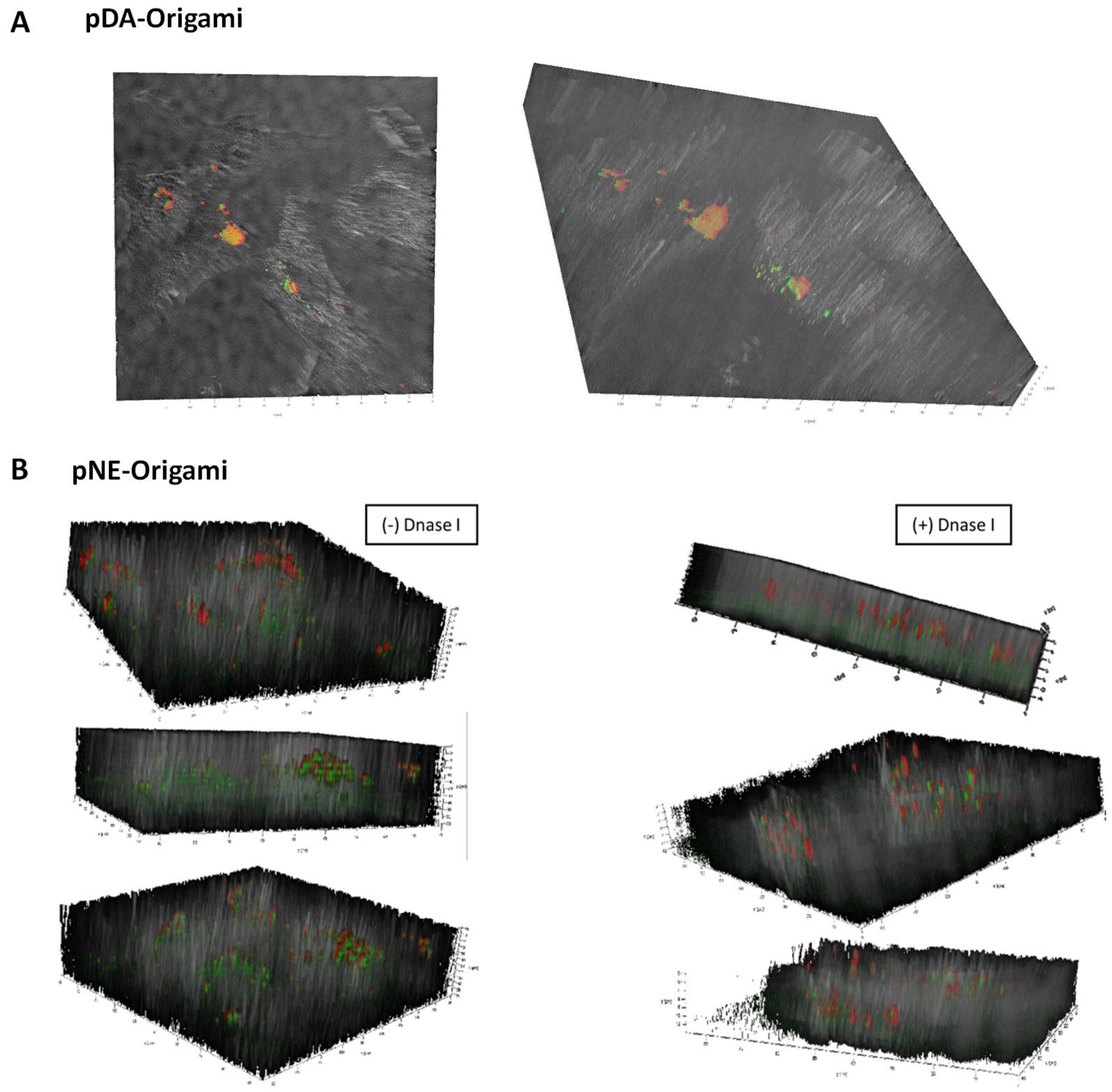

Figure 20 Confocal z-stack analysis followed by 3D reconstruction of pDA- and pNE-origami incubated A549 cells. (A) Z-stacks of the pDA-sample reveal that the observed aggregates are not internalized by the cells (no DNase I treatment). (B) In contrast, pNE-ringed origami tubes are found inside the cells, with and without DNase treatment.

Appendix

DNA Sequences

DNA origami tubes were synthesized together with M13mp18 Scaffold DNA and staple strands according to the above-mentioned protocol. In the following, the sequences of staple strands and additional oligonucleotides are listed.

For G4 staple strands, the sequence of the respective staple strand is extended at the 3' end by 5'-TTTTGGGTAGGGCGGGTTGGG-3'.
For stickyA sequences, the sequence of the respective staple strand is extended at the 3' end by 5'- TTTTTAGTAGGTGGTAGAG-3'.
For stickyC sequences, the sequence of the respective staple strand is extended at the 3' end by 5'- TTTTCTCTCCTTCCCTTT-3'.
G4 complementary strands: 5'- GGGTTGGGCGGGATGGGTTTTCTCTACCACCTACTA-3'
Alexa-488® oligonucleotide: Alexa488-5'- TTCTCTACCACCTACTA-3'
Alexa-647® oligonucleotide: Alexa647-5'-TTAAAGGGAAGGAGAG-3'

To introduce functionalities on the bare DNA origami tube (Scaffold DNA + 206 staple strands + 20 folding strands; Appendix SI Figure 1), respective staple strands were replaced by G4-staple strands or sticky sequences. Staple strands at the long edges of the rectangle were replaced by folding strands.

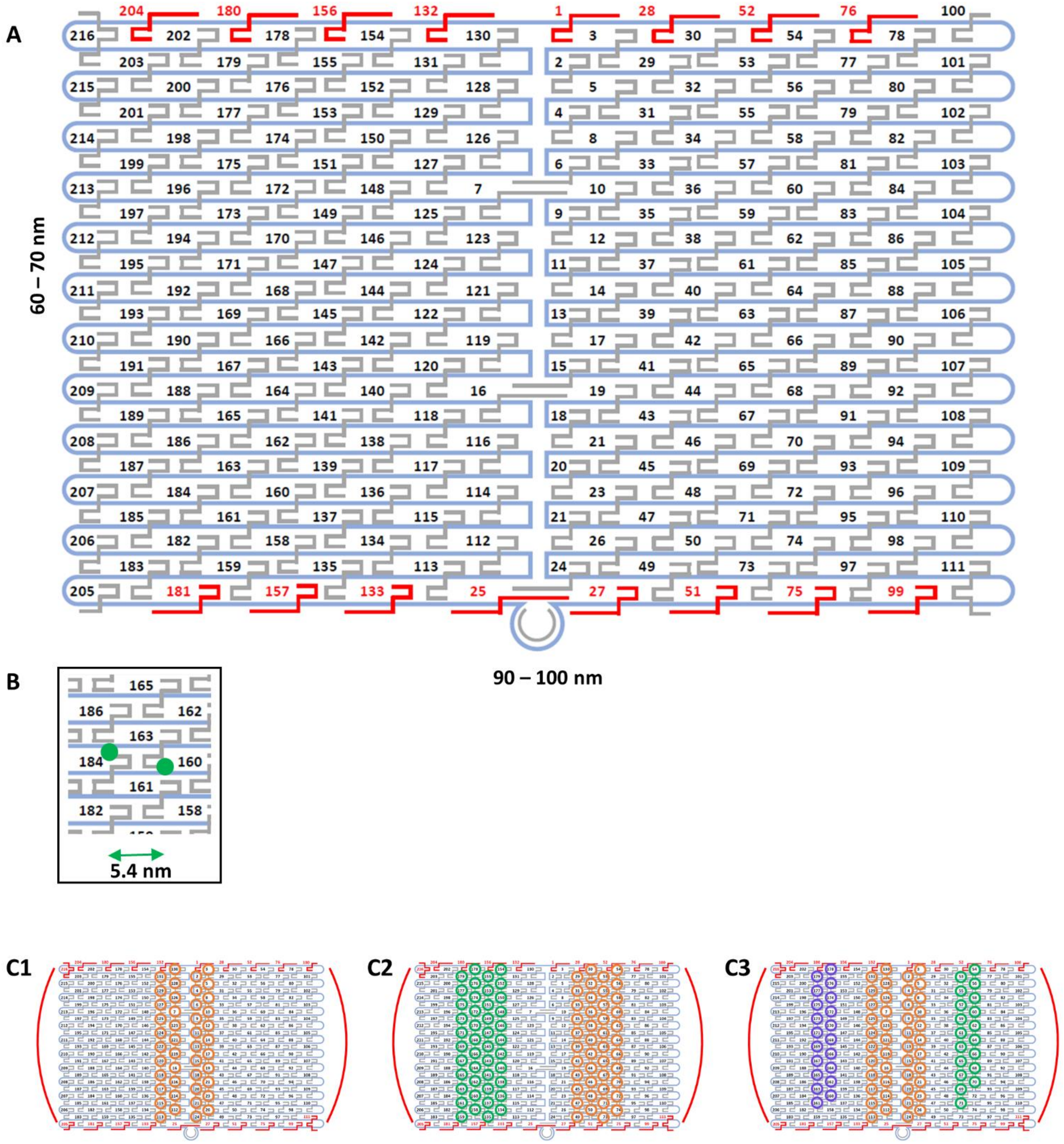

Appendix SI Figure 1 (A) Footprint of a DNA origami rectangle where the position of each staple strand is depicted. By replacing the staple strands at the long edges of the rectangle by folding strands (red), a tube structure is formed. (B) Distance between two attachment points is 5.4 nm. (C) Footprints of the employed structures: (1) tube with 1 central G4 ring (orange), (2) tube with 1 G4 ring and 1 sticky A ring (green), (3) tube for colocalization studies with G4 ring and stickyA and stickyC (purple) for labelling.

Tube with 1 central G4 ring: G4 staple strands on positions 2–10, 11–20, 21–24, 26, 112–120, 121–130, 131; folding strands on positions 1, 25, 27, 28, 51, 52, 75, 76, 99, 100, 111, 132, 133, 156, 157, 180, 181, 204, 205, 216; staple strands on remaining positions.

Tube with 1 G4 ring and 1 stickyA ring at the tubular ends: G4 staple strands on positions 29, 30, 31–40, 41–50, 53–60, 61–70, 71–74; stickyA strands on positions 134–140, 141–150, 151–155, 158–160, 161–170, 171–179; folding strands on positions 1, 25, 27, 28, 51, 52, 75, 76, 99, 100, 111, 132, 133, 156, 157, 180, 181, 204, 205, 216; staple strands on remaining positions.

Tube with 1 central G4 ring and stickyC for dye labelling: G4 staple strands on positions 2–10, 11–20, 21–24, 26, 112–120, 121–130, 131; stickyC strands on positions 161-170, 171-179; folding strands on positions 1, 25, 27, 28, 51, 52, 75, 76, 99, 100, 111, 132, 133, 156, 157, 180, 181, 204, 205, 216; staple strands on remaining positions.

Tube with 1 central G4 ring and stickyC+stickyA for two dye labelling: G4 staple strands on positions 2–10, 11–20, 21–24, 26, 112–120, 121–130, 131; stickyC strands on positions 161-170, 171-179; stickyA strands on positions 53-60, 61-70, 71; folding strands on positions 1, 25, 27, 28, 51, 52, 75, 76, 99, 100, 111, 132, 133, 156, 157, 180, 181, 204, 205, 216; staple strands on remaining positions.

Table S1. Sequences of staple strands and folding strands.

#	Sequence 5' → 3'	#	Sequence 5' → 3'
1	CAAGCCCAATAGGAACCCATGTACAAACAGTT	124	AAGAGGAACGAGCTTCAAAGCGAAGATACATT
2	AATGCCCCGTAACAGTGCCCGTATCTCCCTCA	125	GGAATTACTCGTTTACCAGACGACAAAAGATT
3	TGCCTTGACTGCCTATTTCGGAACAGGGATAG	126	GAATAAGGACGTAACAAAGCTGCTCTAAAACA
4	GAGCCGCCCCACCACCGGAACCGCGACGGAAA	127	CCAAATCACTTGCCCTGACGAGAACGCCAAAA
5	AACCAGAGACCCTCAGAACCGCCAGGGGTCAG	128	CTCATCTTGAGGCAAAAGAATACAGTGAATTT
6	TTATTCATAGGGAAGGTAAATATTCATTCAGT	129	AAACGAAATGACCCCCAGCGATTATTCATTAC
7	CATAACCCGAGGCATAGTAAGAGCTTTTTAAG	130	CTTAAACATCAGCTTGCTTTCGAGCGTAACAC
8	ATTGAGGGTAAAGGTGAATTATCAATCACCGG	131	TCGGTTTAGCTTGATACCGATAGTCCAACCTA
9	AAAAGTAATATCTTACCGAAGCCCTTCCAGAG	132	TGAGTTTCGTCACCAGTACAAACTTAATTGTA
10	GCAATAGCGCAGATAGCCGAACAATTCAACCG	133	CCCCGATTTAGAGCTTGACGGGGAAATCAAAA
11	CCTAATTTACGCTAACGAGCGTCTAATCAATA	134	GAATAGCCGCAAGCGGTCCACGCTCCTAATGA
12	TCTTACCAGCCAGTTACAAAATAAATGAAATA	135	GAGTTGCACGAGATAGGGTTGAGTAAGGGAGC
13	ATCGGCTGCGAGCATGTAGAAACCTATCATAT	136	GTGAGCTAGTTTCCTGTGTGAAATTTGGGAAG
14	CTAATTTATCTTTCCTTATCATTCATCCTGAA	137	TCATAGCTACTCACATTAATTGCGCCCTGAGA
15	GCGTTATAGAAAAAGCCTGTTTAGAAGGCCGG	138	GGCGATCGCACTCCAGCCAGCTTTGCCATCAA
16	GCTCATTTTCGCATTAAATTTTTGAGCTTAGA	139	GAAGATCGGTGCGGGCCTCTTCGCAATCATGG
17	AATTACTACAAATTCTTACCAGTAATCCCATC	140	AAATAATTTTAAATTGTAAACGTTGATATTCA
18	TTAAGACGTTGAAAACATAGCGATAACAGTAC	141	GCAAATATCGCGTCTGGCCTTCCTGGCCTCAG
19	TAGAATCCCTGAGAAGAGTCAATAGGAATCAT	142	ACCGTTCTAAATGCAATGCCTGAGAGGTGGCA
20	CTTTTACACAGATGAATATACAGTAAACAATT	143	TATATTTTAGCTGATAAATTAATGTTGTATAA
21	TTTAACGTTCGGGAGAAACAATAATTTTCCCT	144	TCAATTCTTTTAGTTTGACCATTACCAGACCG
22	CGACAACTAAGTATTAGACTTTACAATACCGA	145	CGAGTAGAACTAATAGTAGTAGCAAACCCTCA
23	GGATTTAGCGTATTAAATCCTTTGTTTTCAGG	146	GAAGCAAAAAAGCGGATTGCATCAGATAAAAA
24	ACGAACCAAAACATCGCCATTAAATGGTGGTT	147	TCAGAAGCCTCCAACAGGTCAGGATCTGCGAA
25	GAACGTGGCGAGAAAGGAAGGGAACAAACTAT	148	CCAAAATATAATGCAGATACATAAACACCAGA
26	TAGCCCTACCAGCAGAAGATAAAAACATTTGA	149	CATTCAACGCGAGAGGCTTTTGCATATTATAG
27	CGGCCTTGCTGGTAATATCCAGAACGAACTGA	150	ACGAGTAGTGACAAGAACCGGATATACCAAGC
28	CTCAGAGCCACCACCCTCATTTTCCTATTATT	151	AGTAATCTTAAATTGGGCTTGAGAGAATACCA
29	CTGAAACAGGTAATAAGTTTTAACCCCTCAGA	152	GCGAAACATGCCACTACGAAGGCATGCGCCGA
30	AGTGTACTTGAAAGTATTAAGAGGCCGCCACC	153	ATACGTAAAAGTACAACGGAGATTTCATCAAG
31	GCCACCACTCTTTTCATAATCAAACCGTCACC	154	CAATGACACTCCAAAAGGAGCCTTACAACGCC
32	GTTTGCCACCTCAGAGCCGCCACCGATACAGG	155	AAAAAAGGACAACCATCGCCCACGCGGGTAAA
33	GACTTGAGAGACAAAAGGGCGACAAGTTACCA	156	TGTAGCATTCCACAGACAGCCCTCATCTCCAA
34	AGCGCCAACCATTTGGGAATTAGATTATTAGC	157	GTAAAGCACTAAATCGGAACCCTAGTTGTTCC
35	GAAGGAAAATAAGAGCAAGAAACAACAGCCAT	158	AGTTTGGAGCCCTTCACCGCCTGGTTGCGCTC
36	GCCCAATACCGAGGAAACGCAATAGGTTTACC	159	AGCTGATTACAAGAGTCCACTATTGAGGTGCC
37	ATTATTTAACCCAGCTACAATTTTCAAGAACG	160	ACTGCCCGCCGAGCTCGAATTCGTTATTACGC
38	TATTTTGCTCCCAATCCAAATAAGTGAGTTAA	161	CCCGGGTACTTTCCAGTCGGGAAACGGGCAAC
39	GGTATTAAGAACAAGAAAAATAATTAAAGCCA	162	CAGCTGGCGGACGACGACAGTATCGTAGCCAG
40	TAAGTCCTACCAAGTACCGCACTCTTAGTTGC	163	GTTTGAGGGAAAGGGGGATGTGCTAGAGGATC
41	ACGCTCAAAATAAGAATAAACACCGTGAATTT	164	CTTTCATCCCCAAAAACAGGAAGACCGGAGAG
42	AGGCGTTACAGTAGGGCTTAATTGACAATAGA	165	AGAAAAGCAACATTAAATGTGAGCATCTGCCA
43	ATCAAAATCGTCGCTATTAATTAACGGATTCG	166	GGTAGCTAGGATAAAAATTTTTAGTTAACATC
44	CTGTAAATCATAGGTCTGAGAGACGATAAATA	167	CAACGCAATTTTTGAGAGATCTACTGATAATC
45	CCTGATTGAAAGAAATTGCGTAGACCCGAACG	168	CAATAAATACAGTTGATTCCCAATTTAGAGAG
46	ACAGAAATCTTTGAATACCAAGTTCCTTGCTT	169	TCCATATACATACAGGCAAGGCAACTTTATTT
47	TTATTAATGCCGTCAATAGATAATCAGAGGTG	170	TACCTTTAAGGTCTTTACCCTGACAAAGAAGT
48	AGATTAGATTTAAAAGTTTGAGTACACGTAAA	171	CAAAAATCATTGCTCCTTTTGATAAGTTTCAT
49	AGGCGGTCATTAGTCTTTAATGCGCAATATTA	172	TTTGCCAGATCAGTTGAGATTTAGTGGTTTAA
50	GAATGGCTAGTATTAACACCGCCTCAACTAAT	173	AAAGATTCAGGGGGTAATAGTAAACCATAAAT
51	CCGCCAGCCATTGCAACAGGAAAAATATTTTT	174	TTTCAACTATAGGCTGGCTGACCTTGTATCAT
52	CCCTCAGAACCGCCACCCTCAGAACTGAGACT	175	CCAGGCGCTTAATCATTGTGAATTACAGGTAG
53	CCTCAAGAATACATGGCTTTTGATAGAACCAC	176	CGCCTGATGGAAGTTTCCATTAAACATAACCG
54	TAAGCGTCGAAGGATTAGGATTAGTACCGCCA	177	TTTCATGAAAATTGTGTCGAAATCTGTACAGA
55	CACCAGAGTTCGGTCATAGCCCCCGCCAGCAA	178	ATATATTCTTTTTTCACGTTGAAAATAGTTAG
56	TCGGCATTCCGCCGCCAGCATTGACGTTCCAG	179	AATAATAAGGTCGCTGAGGCTTGCAAAGACTT
57	AATCACCAAATAGAAAATTCATATATAACGGA	180	CGTAACGATCTAAAGTTTTGTCGTGAATTGCG

58	TCACAATCGTAGCACCATTACCATCGTTTTCA	181	ACCCAAATCAAGTTTTTTGGGGTCAAAGAACG
59	ATACCCAAGATAACCCACAAGAATAAACGATT	182	TGGACTCCCTTTTCACCAGTGAGACCTGTCGT
60	ATCAGAGAAAGAACTGGCATGATTTTATTTTG	183	TGGTTTTTAACGTCAAAGGGCGAAGAACCATC
61	TTTTGTTTAAGCCTTAAATCAAGAATCGAGAA	184	GCCAGCTGCCTGCAGGTCGACTCTGCAAGGCG
62	AGGTTTTGAACGTCAAAAATGAAAGCGCTAAT	185	CTTGCATGCATTAATGAATCGGCCCGCCAGGG
63	CAAGCAAGACGCGCCTGTTTATCAAGAATCGC	186	ATTAAGTTCGCATCGTAACCGTGCGAGTAACA
64	AATGCAGACCGTTTTTATTTTCATCTTGCGGG	187	TAGATGGGGGGTAACGCCAGGGTTGTGCCAAG
65	CATATTTAGAAATACCGACCGTGTTACCTTTT	188	ACCCGTCGTCATATGTACCCCGGTAAAGGCTA
66	AATGGTTTACAACGCCAACATGTAGTTCAGCT	189	CATGTCAAGATTCTCCGTGGGAACCGTTGGTG
67	TAACCTCCATATGTGAGTGAATAAACAAAATC	190	TCAGGTCACTTTTGCGGGAGAAGCAGAATTAG
68	AAATCAATGGCTTAGGTTGGGTTACTAAATTT	191	CTGTAATATTGCCTGAGAGTCTGGAAAACTAG
69	GCGCAGAGATATCAAAATTATTTGACATTATC	192	CAAAATTAAAGTACGGTGTCTGGAAGAGGTCA
70	AACCTACCGCGAATTATTCATTTCCAGTACAT	193	TGCAACTAAGCAATAAAGCCTCAGTTATGACC
71	ATTTTGCGTCTTTAGGAGCACTAAGCAACAGT	194	TTTTTGCGCAGAAAACGAGAATGAATGTTTAG
72	CTAAAATAGAACAAAGAAACCACCAGGGTTAG	195	AAACAGTTGATGGCTTAGAGCTTATTTAAATA
73	GCCACGCTATACGTGGCACAGACAACGCTCAT	196	ACTGGATAACGGAACAACATTATTACCTTATG
74	GCGTAAGAGAGAGCCAGCAGCAAAAAGGTTAT	197	ACGAACTAGCGTCCAATACTGCGGAATGCTTT
75	GGAAATACCTACATTTTGACGCTCACCTGAAA	198	CGATTTTAGAGGACAGATGAACGGCGCGACCT
76	TATCACCGTACTCAGGAGGTTTAGCGGGGTTT	199	CTTTGAAAAGAACTGGCTCATTATTTAATAAA
77	TGCTCAGTCAGTCTCTGAATTTACCAGGAGGT	200	GCTCCATGAGAGGCTTTGAGGACTAGGGAGTT
78	GGAAAGCGACCAGGCGGATAAGTGAATAGGTG	201	ACGGCTACTTACTTAGCCGGAACGCTGACCAA
79	TGAGGCAGGCGTCAGACTGTAGCGTAGCAAGG	202	AAAGGCCGAAAGGAACAACTAAAGCTTTCCAG
80	TGCCTTTAGTCAGACGATTGGCCTGCCAGAAT	203	GAGAATAGCTTTTGCGGGATCGTCGGGTAGCA
81	CCGGAAACACACCACGGAATAAGTAAGACTCC	204	ACGTTAGTAAATGAATTTTCTGTAAGCGGAGT
82	ACGCAAAGGTCACCAATGAAACCAATCAAGTT	205	TTTTCGATGGCCCACTACGTAAACCGTC
83	TTATTACGGTCAGAGGGTAATTGAATAGCAGC	206	TATCAGGGTTTTCGGTTTGCGTATTGGGAACGCGCG
84	TGAACAAACAGTATGTTAGCAAACTAAAAGAA	207	GGGAGAGGTTTTTGTAAAACGACGGCCATTCCCAGT
85	CTTTACAGTTAGCGAACCTCCCGACGTAGGAA	208	CACGACGTTTTTGTAATGGGATAGGTCAAAACGGCG
86	GAGGCGTTAGAGAATAACATAAAAGAACACCC	209	GATTGACCTTTTGATGAACGGTAATCGTAGCAAACA
87	TCATTACCCGACAATAAACAACATATTTAGGC	210	AGAGAATCTTTTGGTTGTACCAAAAACAAGCATAAA
88	CCAGACGAGCGCCCAATAGCAAGCAAGAACGC	211	GCTAAATCTTTTCTGTAGCTCAACATGTATTGCTGA
89	AGAGGCATAATTTCATCTTCTGACTATAACTA	212	ATATAATGTTTTCATTGAATCCCCCTCAAATCGTCA
90	TTTTAGTTTTTCGAGCCAGTAATAAATTCTGT	213	TAAATATTTTTTGGAAGAAAAATCTACGACCAGTCA
91	TATGTAAACCTTTTTTAATGGAAAAATTACCT	214	GGACGTTGTTTTTCATAAGGGAACCGAAAGGCGCAG
92	TTGAATTATGCTGATGCAAATCCACAAATATA	215	ACGGTCAATTTTGACAGCATCGGAACGAACCCTCAG
93	GAGCAAAAACTTCTGAATAATGGAAGAAGGAG	216	CAGCGAAAATTTTACTTTCAACAGTTTCTGGGATTTTGCTAAACTTTT
94	TGGATTATGAAGATGATGAAACAAAATTTCAT	217	AACATCACTTGCCTGAGTAGAAGAACT
95	CGGAATTATTGAAAGGAATTGAGGTGAAAAAT	218	TGTAGCAATACTTCTTTGATTAGTAAT
96	ATCAACAGTCATCATATTCCTGATTGATTGTT	219	AGTCTGTCCATCACGCAAATTAACCGT
97	CTAAAGCAAGATAGAACCCTTCTGAATCGTCT	220	ATAATCAGTGAGGCCACCGAGTAAAAG
98	GCCAACAGTCACCTTGCTGAACCTGTTGGCAA	221	ACGCCAGAATCCTGAGAAGTGTTTTT
99	GAAATGGATTATTTACATTGGCAGACATTCTG	222	TTAAAGGGATTTTAGACAGGAACGGT
100	TTTTTATAAGTATAGCCCGGCCGTCGAG	223	AGAGCGGGAGCTAAACAGGAGGCCGA
101	AGGGTTGATTTTATAAATCCTCATTAAATGATATTC	224	TATAACGTGCTTTCCTCGTTAGAATC
102	ACAAACAATTTTAATCAGTAGCGACAGATCGATAGC	225	GTACTATGGTTGCTTTGACGAGCACG
103	AGCACCGTTTTTTAAAGGTGGCAACATAGTAGAAAA	226	GCGCTTAATGCGCCGCTACAGGGCGC
104	TACATACATTTTGACGGGAGAATTAACTACAGGGAA	F1	CGGCCTTGATAGGAACCCATGTACAAACAGTT
105	GCGCATTATTTTGCTTATCCGGTATTCTAAATCAGA	F25	TGAGTTTCCGAGAAAGGAAGGGAACAAACTAT
106	TATAGAAGTTTTCGACAAAAGGTAAAGTAGAGAATA	F27	CAAGCCCACTGGTAATATCCAGAACGAACTGA
107	TAAAGTACTTTTCGCGAGAAAACTTTTTATCGCAAG	F28	CCGCCAGCCACCACCCTCATTTTCCTATTATT
108	ACAAAGAATTTTATTAATTACATTTAACACATCAAG	F51	CTCAGAGCCATTGCAACAGGAAAAATATTTTT
109	AAAACAAATTTTTTCATCAATATAATCCTATCAGAT	F52	GGAAATACACCGCCACCCTCAGAACTGAGACT
110	GATGGCAATTTTAATCAATATCTGGTCACAAATATC	F75	CCCTCAGACTACATTTTGACGCTCACCTGAAA
111	AAACCCTCTTTTACCAGTAATAAAAGGGATTCACCAGTCACACGTTTT	F76	GAAATGGATACTCAGGAGGTTTAGCGGGGTTT
112	CCGAAATCCGAAAATCCTGTTTGAAGCCGGAA	F99	TATCACCGTTATTTACATTGGCAGACATTCTG
113	CCAGCAGGGGCAAAATCCCTTATAAAGCCGGC	F132	GAACGTGGGTCACCAGTACAAACTTAATTGTA
114	GCATAAAGTTCCACACAACATACGAAGCGCCA	F133	TGTAGCATTAGAGCTTGACGGGGAAATCAAAA
115	GCTCACAATGTAAAGCCTGGGGTGGGTTTGCC	F156	CCCCGATTTCCACAGACAGCCCTCATCTCCAA
116	TTCGCCATTGCCGGAAACCAGGCATTAAATCA	F157	CGTAACGACTAAATCGGAACCCTAGTTGTTCC
117	GCTTCTGGTCAGGCTGCGCAACTGTGTTATCC	F180	GTAAAGCATCTAAAGTTTTGTCGTGAATTGCG
118	GTTAAAATTTTAACCAATAGGAACCCGGCACC	F181	ACGTTAGTCAAGTTTTTTGGGGTCAAAGAACG
119	AGACAGTCATTCAAAAGGGTGAGAAGCTATAT	F204	ACCCAAATAAATGAATTTTCTGTAAGCGGAGT
120	AGGTAAAGAAATCACCATCAATATAATATTTT	F100	GTCACACGTTTTTATAAGTATAGCCCGGCCGTCGAG
121	TTTCATTTGGTCAATAACCTGTTTATATCGCG	F205	TGCTAAACTTTTCGATGGCCCACTACGTAAACCGTC
122	TCGCAAATGGGGCGCGAGCTGAAATAATGTGT	N-111	AAACCCTCTTTTACCAGTAATAAAAGGGATTCACCA
123	TTTTAATTGCCCGAAAGACTTCAAAACACTAT	N-216	CAGCGAAATTTTAACTTTCAACAGTTTCTGGGATTT

References

[1] E. Stahl, T. G. Martin, F. Praetorius, H. Dietz, *Angewandte Chemie* **2014**, *126*, 12949-12954.
[2] (a) H. Lee, S. M. Dellatore, W. M. Miller, P. B. Messersmith, *Science* **2007**, *318*, 426-430; (b) D. R. Dreyer, D. J. Miller, B. D. Freeman, D. R. Paul, C. W. Bielawski, *Langmuir* **2012**, *28*, 6428-6435; (c) S. Hong, Y. S. Na, S. Choi, I. T. Song, W. Y. Kim, H. Lee, *Advanced Functional Materials* **2012**, *22*, 4711-4717.
[3] (a) P. Manini, A. Pezzella, L. Panzella, A. Napolitano, M. d'Ischia, *Tetrahedron* **2005**, *61*, 4075-4080; (b) P. Manini, L. Panzella, A. Napolitano, M. d'Ischia, *Chem Res Toxicol* **2007**, *20*, 1549-1555; (c) S. Hong, J. Kim, Y. S. Na, J. Park, S. Kim, K. Singha, G. I. Im, D. K. Han, W. J. Kim, H. Lee, *Angew Chem Int Ed Engl* **2013**, *52*, 9187-9191; (d) X. Tan, P. Gao, Y. Li, P. Qi, J. Liu, R. Shen, L. Wang, N. Huang, K. Xiong, W. Tian, Q. Tu, *Bioact Mater* **2021**, *6*, 285-296.

Author Contributions

P. W. conducted all the DNA origami experiments and analysis. C. J. W. and D.Y.W.N. conducted cell experiments and confocal microscopy analysis. The project was supervised by D.Y.W.N. and T.W. All authors contributed to writing the manuscript.
